# Austenite Grain Growth Analysis in a Welded Joint of High-Strength Martensitic Abrasion-Resistant Steel Hardox 450

**DOI:** 10.3390/ma14112850

**Published:** 2021-05-26

**Authors:** Łukasz Konat, Martyna Zemlik, Robert Jasiński, Dominika Grygier

**Affiliations:** Department of Vehicle Engineering, Wroclaw University of Science and Technology, 50-370 Wroclaw, Poland; lukasz.konat@pwr.edu.pl (Ł.K.); robert.jasinski@pwr.edu.pl (R.J.); dominika.grygier@pwr.edu.pl (D.G.)

**Keywords:** wear-resistant martensitic steel, submerged arc welding (SAW), structures, mechanical properties, prior austenite grain size, Hardox 450 steel

## Abstract

The paper presents the results of tests of a welded joint of Hardox 450 steel, belonging to the group of weldable high-strength boron steels with increased resistance to abrasive wear. As a result of the conducted research, apart from the basic structural indicators, an attempt was made to determine the correlation between the grain size of the prior austenite in the characteristic weld zones and its basic mechanical properties, such as yield point, tensile strength, percentage elongation after fracture, reduction of area, and impact strength. The scope of research quoted above was carried out for a welded joint of the considered steel at delivery state (directly after welding), in the normalising annealed state, as well as in water-quenched state, using different austenitisation temperatures in the range of 900–1200 °C. The results obtained showed a large influence of the parameters of the applied thermal heat treatment on the selected structural and mechanical properties of the welded joint.

## 1. Introduction

Hardox steels belong to the most widespread group of low-alloy steels resistant to abrasive wear with boron. According to generally available information [1], they are characterised by very high strength properties, resistance to impact loads, machinability, and good weldability, which is especially emphasised by the manufacturer. The following types, classified based on average hardness in the Brinell scale, can be distinguished among the commercially offered Hardox steels: Hardox 400, Hardox 450, Hardox 500, Hardox 500 Tuf, Hardox 550, and Hardox 600. The above list can also serve as a criterion for the weldability of Hardox steels, which are systematised in the descending order. Apart from the classification presented above, there are also other types of the discussed material group, i.e., Hardox HiTuff (high-impact strength), HiTemp (high temperature-resistant) and Hardox Extreme, which is described as the world’s hardest commercially available plate. Extensive analyses of available information materials [1,2] and numerous own tests, e.g., [3,4,5,6,7,8,9,10,11,12], make it possible to state that all of the steels mentioned above are delivered in the as-quenched state and, in the case of the required hardness level, also tempering in the range of 200–700 °C is carried out. Furthermore, it should be mentioned that in each of the discussed cases, the chemical properties quoted by the manufacturer include only the maximum values of particular alloying elements. This is caused by quite a large variability of the chemical composition, resulting from a wide range of available plate thicknesses (in the case of Hardox 400, i.e., 4–130 mm), while maintaining a homogeneous structure with post-martensitic orientation across the entire cross-section of the offered steel products. Table 1, Table 2 and Table 3 shows selected chemical and mechanical properties of Hardox 450 steel, considering the available plate thicknesses.

Based on the data summarised in Table 1 and Table 2, it can be generally stated that in the case of Hardox 450 for the plate thickness range 10–40 mm, the carbon content can vary within 0.17–0.26%, chromium 0.45–1.40%, manganese 1.00–1.60%, nickel 0.05–1.50%, molybdenum 0.08–0.60%, silicon 0.32–0.70%, and boron within 0.0014–0.005%. As previously mentioned, the reason for the large variation in chemical composition is the need to obtain a homogeneous structure, as well as a similar level of hardness across the plate cross-section. From the metallurgical process point of view, these properties are realised by an appropriate quenching level (addition of boron) and, if necessary (increased plate thickness), by increasing the proportion of Cr, Mn, and Ni. For the steel under consideration, tempering processes in the temperature range 250–400 °C are not excluded either, which implies the need to prevent irreversible temper brittleness by the addition of Mo, above 0.20%. Therefore, the manufacturing process of Hardox 450 (and other steels) must involve a high degree of individualisation of the heat treatment processes, which, especially in the welded joints of the steel, must result in variations in structure and performance properties.

Regardless of the above-mentioned considerations, a very important criterion from the point of view of practical applicability of Hardox 450 is its weldability. In relation to other steels, i.e., Hardox 400 and Hardox 500, the metallurgical weldability parameter has already been verified, e.g., [10,13]. As a result of the work carried out, it can be generally stated that these steels are characterised by good (Hardox 400) and satisfactory (Hardox 500) weldability. However, in each case, welding operations led to large structural changes within the broad heat-affected zone, resulting in varied hardness changes, as well as a local loss of abrasive wear resistance. Furthermore, it should be noted that the phenomena described above were accompanied by a very large reduction in strength indices and a radical decrease in break work. Therefore, from a practical point of view, the issues of structural changes occurring during the welding process and post-heat treatment of welded joints of selected Hardox steels seem to be worth considering. Since in relation to the original material (Hardox 450), the authors of this study have already carried out described structural analyses (Table 3) [4], the presented work is a comprehensive extension of this subject, and it is focused on the issues related to the influence of temperature on representative zones of welded joints of the aforesaid steel.

One of the primary ways to increase the strength properties of steel is to strengthen it with grain borders. This relationship was defined by Hall–Petch (1) and (2), whereby, at ambient temperature, the increase in most strength indices can be associated with fragmentation (reduction in grain size) of the structure [14,15,16,17]. A similar relation can also be formulated with respect to destruction of steel as a result of brittle fracture (3) [18]. The assumption is that an increase in the size of the grain forming material structures facilitates the fracture process, as the greater the number of accumulated dislocations, the lower stress is sufficient for a fracture to develop.

(1) $$\sigma_{y}=\sigma_{0}+\frac{k_{y}}{\sqrt{d}},$$

where

σ_y_ is the yield point;
σ_0_ is the friction resistance for dislocation movement within the polycrystalline grain;
k_y_ is consolidation coefficient—a measure of the local stress needed at a grain boundary for the transmission of plastic flow—unpinning constant;
d is an average grain size

(2) $$k_{y}\text{\textasciitilde{}}3{\left[\frac{{2\mathrm{Gb}\tau }_{b}}{q\pi }\right]}^{1/2},$$

where

G is the shear modulus;
τ_b_ is the Burgers vector (dependent on the type of crystal lattice);
q is the geometric factor (dependent on lattice type);
b is the critical stress required to transmit sliding through grains boundary

(3) $$\sigma_{f}=\sigma_{0f}\frac{k_{f}}{\sqrt{d}},$$

where

σ_f_ is the material strength;
σ_0f_ and k_f_ are experimentally determined constants, for k_f_ > k_y_

(4) $$k_{f}\geq {\left(\frac{6\pi \gamma G}{1-\nu }\right)}^{2},$$

where

γ is the fracture surface energy;
ν is the Poisson’s ratio.

The size of the prior austenite grain also defines the properties of martensitic steels. Steels whose martensite morphology is characterised by a lath structure exhibit high strength parameters [19]. The ordering of such a structure with respect to a prior austenite grain can be represented hierarchically, where several packets are formed in a single prior austenite grain, each with a different habitus plane. The packets can be further subdivided into parallel blocks, i.e., areas that share the same crystallographic orientation, which in turn are subdivided into laths [20]. The lath forms the basic crystallographic arrangement of martensite. According to Kurdjumov–Sachs theory, 24 variants of crystallographic orientation of laths are possible [21], because during the shearing process, one of the six {101} surfaces of martensite aligns parallel to one of the four {111} planes of austenite [22]. Based on the available studies, it is believed that both packet and block size affect the strength properties and fracture toughness of steel [14,23]. The relationship between brittle fracture and packet size in martensitic steels has also been studied in [19], where it was proven that packet boundaries can provide a strong obstacle to dislocation movement, and that fragmentation of the microstructure increases the offset yield strength and thus the ultimate strength. There are also first studies on the effect of prior austenite grain size on the abrasive wear resistance of high-strength martensitic steels [24] and on the mechanical properties of martensitic steels alloyed with boron, which, when added in an amount as low as 0.0015%, rapidly increases the quenchability [4,12]. It is worth noting that few studies have been conducted on the effect of boron alloying on the hardening and microstructure of martensitic steels, and those available indicate that its addition implies fragmentation and reduction in the size of bainite laths [25]. Based on this, the decision was made to analyse the effect of the grain size of the prior austenite on the strength properties of the welded joint of the martensitic Hardox 450 steel, which is characterised by increased resistance to abrasive wear and boron micro-addition. An additional reason for taking up this issue are the technological problems encountered during the welding process of this material, in many cases requiring the implementation of post heat treatment.

Based on the manufacturer’s data [1,2] and the results of our own tests, Hardox 450 steel in the delivery state reaches a minimum tensile strength of 1400 MPa (Table 3). Additional strength analyses performed on this material indicate that an increase in the austenitising temperature does not significantly affect tensile strength reduction. It has been shown that austenitising before quenching at 1200 °C results in a value of R_m_ = 1382 MPa, which is only about 50 MPa lower than the delivery state (Table 3). However, the paper [4] indicates that the austenitising temperature constitutes a critical parameter determining the impact properties of Hardox 450. The applied annealing temperature of 1200 °C caused a decrease in impact strength from 70.3 to 19.0 J/cm^2^ in the examined steel.

As far as welded joints are concerned, despite the declared good weldability of Hardox 450 steel, in many cases, it is indicated that the processes involved result in a significant decrease in mechanical properties, resistance to abrasive wear and, what seems to be the most significant, a significant degradation of performance properties of the examined material. The conducted research [13,26] indicates that it is possible to reduce the negative structural changes caused by the welding process by carrying out an appropriate heat treatment. Therefore, the authors take the view that in order to optimise welding processes, it seems reasonable to carry out a meaningful analysis of the structural changes occurring in all zones of a welded joint of Hardox 450 steel during austenitisation in a wide range of temperatures.

## 2. Material and Research Methodology

The research was carried out on steel sheets having the following dimensions: 1000 × 200 mm and 10 mm thick, taken from a sheet of Hardox 450 steel, characterised by the mechanical properties and chemical composition summarised in Table 1, Table 2 and Table 3. Cutting of the sheets to the required dimensions was performed using a HyPerformance numerically controlled plasma system. Welded joints were made by the submerged arc welding (SAW) method, using welding materials dedicated to low-alloy high-strength steels (Table 4). The welding process was carried out with an ESAB A2 Mini Trac welding machine with an ESAB LAE 800 power source (ESAB AB, Göteborg, Sweden). The Hardox plates were joined in a double-welded joint using the following parameters to ensure the plates were properly melted:

- Weld type: BW (butt weld);
- Welding position: PA (flat position);
- Electrode diameter: 3.0 mm;
- Arc voltage (weld 1/2): 31/33 V;
- Current (weld No. 1): 530 A;
- Current (weld No. 2): 630 A;
- Polarity: DC (+);
- Welding speed: ~10 mm/s;
- Electrode wire: OK Autrod 13.43 (S_3_Ni_2.5_CrMo according to EN ISO 26304);
- Flux: OK Flux 10.62 (MgO, CaF_2_, Al_2_O_3_, SiO_2_);
- Preheating: none;
- Interpass temperature: ≤80 °C;
- Preparation of plate edges for welding (bevelling): none.

**Figure 1:**
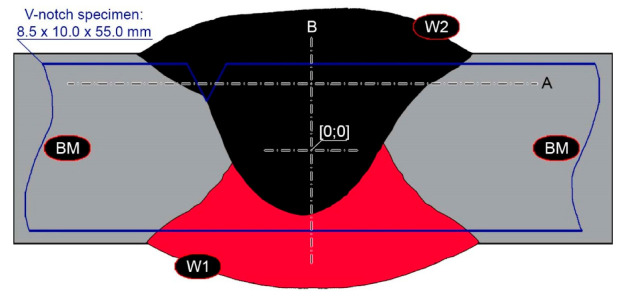
General diagram of a welded joint of Hardox 450. W1/W2—individual layers of the weld in accordance with the order of completion, A/B—lines of the executed hardness distributions, BM—parent material, the place of the V—shaped notch cut on the specimens for impact testing is schematically shown in blue.

Before welding, the positions of the plates were fixed by their permanent restraint to the backing material with positional welds. During welding, run-off plates made of Hardox 450 were used. A diagram of the completed welded joint is shown in Figure 1.

After welding, cuboid-shaped specimens were cut from the plates using high-energy abrasive liquid jet and electroerosion. In subsequent technological operations, some of the welded joint samples were subjected to heat treatment procedures, consisting of volumetric quenching in water and low tempering. Before quenching, the samples underwent an additional normalising annealing. The heat treatments were carried out in Czylok’s FCF 12SHM/R gas-tight chamber furnaces (CZYLOK Company, Jastrzębie-Zdrój, Poland), using a protective atmosphere of neutral gas, 99.95% argon. The quenching bath was performed in de-oxygenated water at a temperature not exceeding 30 °C. Table 5 shows detailed characteristics of the specimens used in the research and the parameters of the performed thermal treatments. With respect to required dimension and roughness tolerances of the surface, at the final stage all specimens were grinded.

Chemical composition analyses were performed using the spectral method with a Leco GDS500A glow discharge spectrometer (LECO Corporation, St. Joseph, MI, USA). The following parameters were applied during the analyses to allow ionisation of the inert gas: U = 1250 V, I = 45 mA, 99.999% argon. The obtained results were the arithmetic average of at least five measurements.

The hardness measurements of the parent material were performed with a Zwick/Roel ZHU universal hardness tester (Zwick Roell Gruppe, Ulm, Germany) using the Brinell method, in accordance with PN-EN ISO 6506-1:2014-12. A cemented carbide ball with a diameter of 2.5 mm was used, with a load of 187.5 kgf (1838.7469 N) applied for 15 s. The hardness measurements on selected cross-sections of welded joints were performed using the Rockwell method (HRA) according to PN-EN ISO 6508–1:2016–10, using the above-mentioned universal hardness tester, applying a load of 60 kgf (588.399 N). The obtained hardness indices were converted to the Vickers scale in accordance with PN-EN ISO 18265:2014–02. The hardness measurements were carried out on cross-sections of the samples in the state after welding and after heat treatment procedures. The locations of the performed hardness distributions are schematically marked with lines A and B in Figure 1.

Macroscopic observations were made with a multifunctional stereo microscope, Nikon AZ100 (Nikon Corporation, Tokyo, Japan). Microstructure was observed using a light microscope, Nikon Eclipse MA200, applying magnifications of 25–1000×. The samples were examined in the non-etched state and after etching with 3% HNO_3_ solution and Adler’s reagent, according to the PN-H-04503:1961 standard. Nikon DS-Fi2 digital cameras (Nikon Corporation, Tokyo, Japan) connected to the microscopes and Nikon NIS Elements software (Nikon Corporation, Tokyo, Japan) were used for recording and analysis of the recorded images.

For the purpose of austenite grain growth analysis, selected samples in the normalised state were austenitised at 900, 1000, 1100, and 1200 °C for 60–120 min, and then subjected to isothermal cooling at 650 °C for 5–10 min. Once the temperature had stabilised throughout the whole volume of the samples, further cooling was carried out in open air. The detailed characteristics of the applied heat treatment variants are listed in Table 5. After preparation of metallographic samples, a 3% solution of HNO_3_ was used for etching. Detailed information on the applied time–temperature parameters is presented in Table 5 and directly under individual microphotographs. The above-mentioned course of action resulted from the presence of numerous alloying micro-additives in the chemical composition of Hardox 450 steel, inhibiting the formation of carbide phases at the grain boundaries of prior austenite or causing a very significant increase in hardenability (boron micro-additives) by blocking diffusion transformations both in the parent and the weld metal. The described situation particularly occurred at the highest austenitising temperatures. The method of revealing the grain boundaries of ferrite and colonies of pearlite (as an equivalents of a prior austenite grain boundaries) was realised according to the PN-EN ISO 643:2020-07 standard. Grain size measurements were performed with the use of ImageJ ver. 1.52a image analysis software developed for the National Institutes of Health. In each case considered, the average grains size of ferrite and pearlite colonies (hereinafter referred to as the prior austenite grains) was determined for 150 measurements.

The tensile tests were carried out at ambient temperature on rectangular, proportional specimens with length L_0_ equal to 5 times the original cross-sectional area S_0_ as per PN-EN ISO 6892-1:2020–05. A material testing system MTS 810 was used with an extensometer having measurement base length L_0_ = 25 mm. During the tests, the stretching rate was controlled by the stress increase rate. Next, the following were determined for each sample: the tensile strength (R_m_), as well as the percentage reduction of area (Z) and percentage elongation after fracture (A_5_). The strength indices were the arithmetic average of the results obtained from at least five samples per measurement point. Additionally, measurement errors were calculated in the form of standard deviation based on the obtained test results for individual samples.

The impact tests were performed for selected sets of samples of welded joints using a Charpy Zwick Roell RPK300 hammer, applying an initial energy value of 300 J, according to the PN-EN ISO 148–1:2017–02 standard. The samples used in the tests were cuboidal with V-shaped notches. The sampling method and the position of the notches in relation to the weld are schematically shown in Figure 1. Based on the test results carried out at temperatures of +20 °C and −40 °C, the arithmetic averages—of at least 5 samples per measuring point—and the standard deviations of the obtained impact indices were determined.

**Figure 2:**
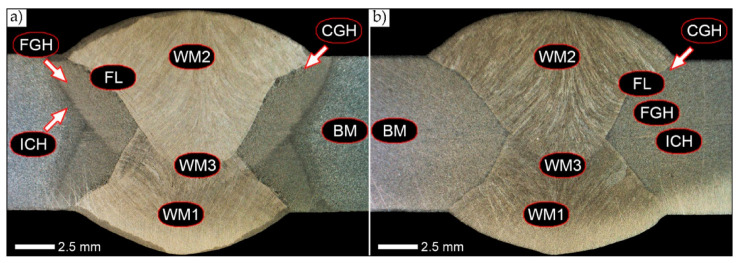
Macroscopic cross-sectional image of a welded joint of Hardox 450 steel: (**a**) delivery state (directly after welding), (**b**) normalised state. WM—weld metal zone, FL—fusion line, BM—parent metal zone, CGH(AZ)—coarse grain zone (overheating), FGH(AZ)—fine grain zone (normalisation and recrystallisation), ICH(AZ)—intercritical zone (incomplete normalisation), HAZ—heat-affected zone. Stereoscopic microscopy, etched with 3% HNO_3_ and Adler’s reagent.

Figure 2 presents macroscopic image of a welded joint of Hardox 450 steel, both in a delivery state and after normalisation. In the first case considered, all specific areas of a heat-affected zone are distinguishable, whereas normalisation results in a structure homogenization. All zones are part of our consideration and performed measurements.

## 3. Results

### 3.1. Hardness Masurements and Microstructure Observations

**Figure 3:**
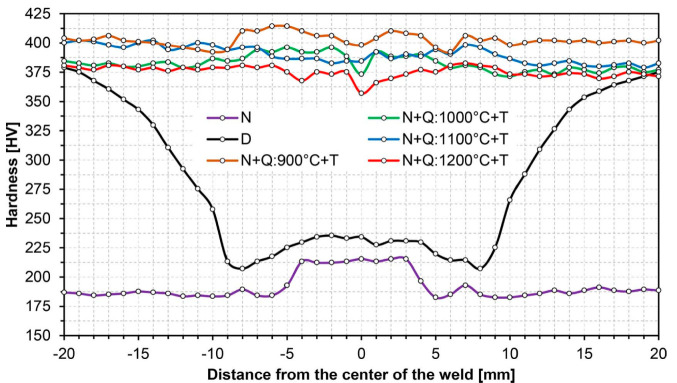
Hardness distributions of the welded joint of Hardox 450 along line A in Figure 1: D—delivery state (directly after welding), N—normalised state, Q—quenched state, T—tempering.

**Figure 4:**
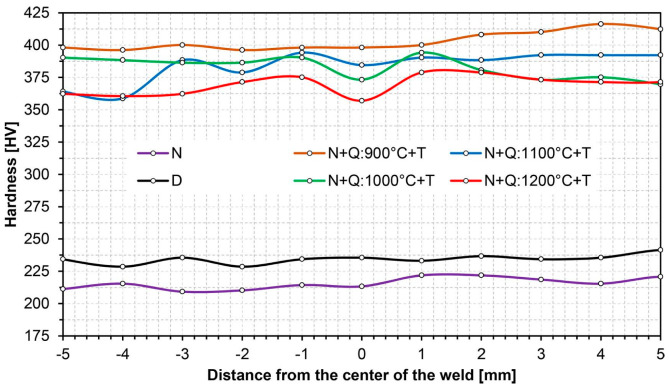
Hardness distributions of the welded joint of Hardox 450 along line B in Figure 1: D—delivery state (directly after welding), N—normalised state, Q—quenched state, T—tempering.

**Figure 5:**
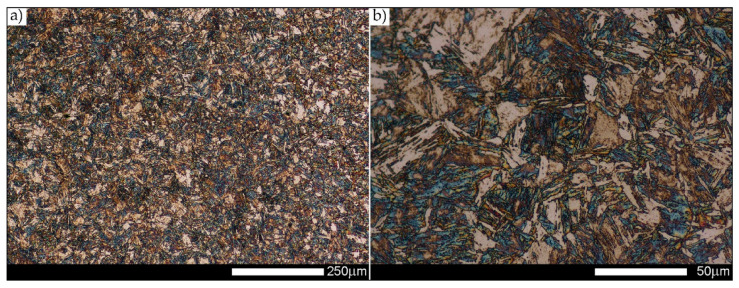
Microstructure of a welded joint of Hardox 450 steel in delivery state (directly after welding)—BM area shown in Figure 2a. (**a**) 100×; (**b**) 500×. Microstructure of low carbon quenching martensite with areas of tempering martensite. Light microscopy, etched with 3% HNO_3_.

**Figure 6:**
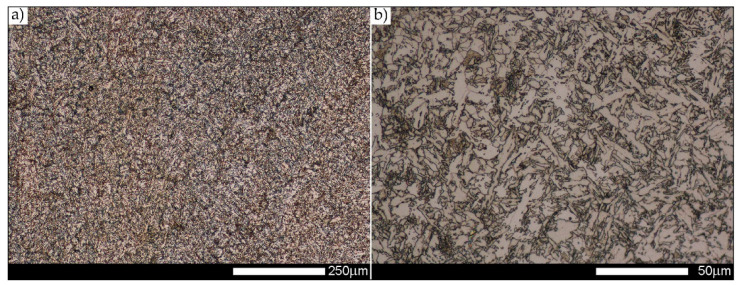
Microstructure of welded joint of Hardox 450 steel in delivery state (directly after welding)—WM1 zone, shown in Figure 2a. (**a**) 100×; (**b**) 500×. Structure of non-equilibrium ferrite grains with Widmanstätten structure features with troostite precipitates. Light microscopy, etched with 3% HNO_3_.

**Figure 7:**
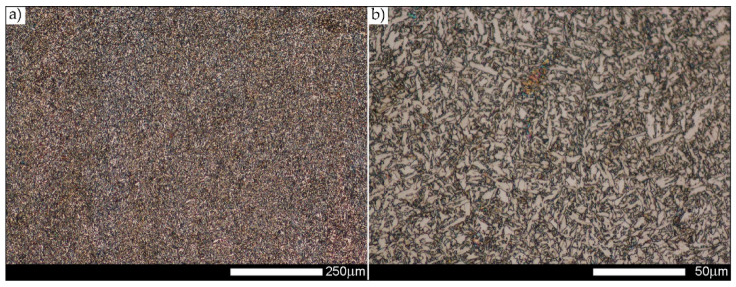
Microstructure of welded joint of Hardox 450 steel in the delivery state (directly after welding)—WM2 zone shown in Figure 2a. (**a**) 100×; (**b**) 500×. Structure of non-equilibrium ferrite grains with troostite precipitates. Light microscopy, etched with 3% HNO_3_.

**Figure 8:**
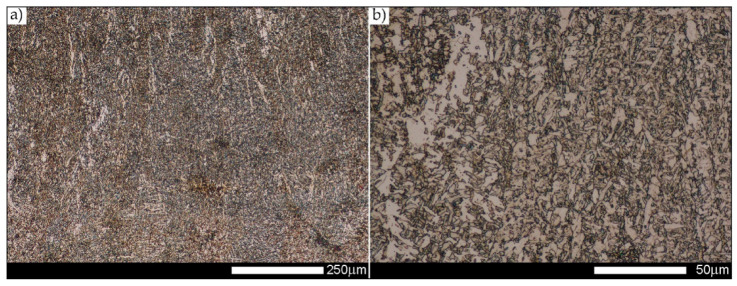
Microstructure of welded joint of Hardox 450 steel in delivery state (directly after welding)—WM3 zone shown in Figure 2a. (**a**) 100×; (**b**) 500×. Structure of non-equilibrium ferrite grains with very clear features of directional crystallisation. Light microscopy, etched with 3% HNO_3_.

**Figure 9:**
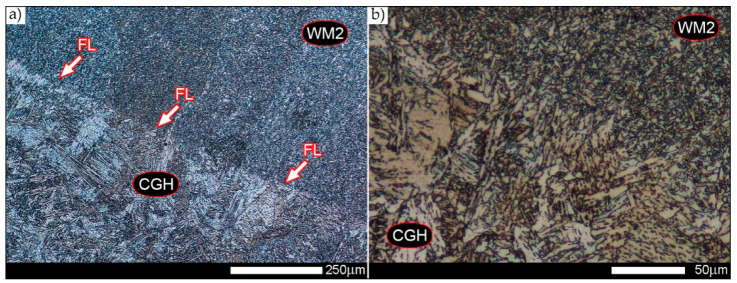
Microstructure of a welded joint of Hardox 450 in delivery state (directly after welding)—FL zone shown in Figure 2a. (**a**) 100×; (**b**) 500×. Clearly visible—narrow—fusion line, mainly consisting of sorbite and troostite structures. A very clear microstructure growth can be observed directly under the fusion line. FL—fusion line, WM2—weld metal zone, CGH(AZ)—coarse grain zone. Light microscopy, etched with 3% HNO_3_.

**Figure 10:**
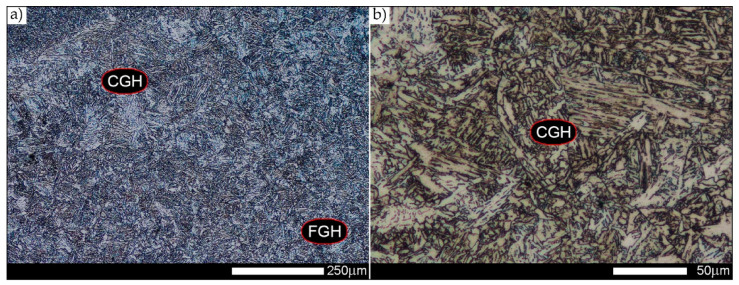
Microstructure of welded joint of Hardox 450 steel in delivery state (directly after welding)—CGH(HAZ) zone shown in Figure 2a. (**a**) 100×; (**b**) 500×. Diversified structure consisting of sorbite and locally quenching martensite. The martensite shows a packet structure with the same crystallographic orientation of the formed blocks within the prior austenite grain boundaries. CGH(AZ)—coarse-grained zone, FGH(AZ)—fine-grained zone. Light microscopy, etched with 3% HNO_3_.

**Figure 11:**
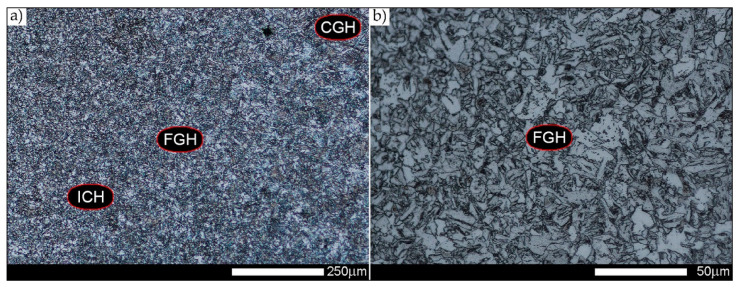
Microstructure of welded joint of Hardox 450 steel in the delivery state (directly after welding)—FGHAZ zone shown in Figure 2a. (**a**) 100×; (**b**) 500×. Fine-grained, morphologically diversified structure consisting of quenching martensite, quenching sorbite and single (small size) colonies of quenching troostite. CGH(AZ)—coarse-grained zone, FGH(AZ)—fine-grained zone, ICH(AZ)—intercritical zone. Light microscopy, etched with 3% HNO_3_.

**Figure 12:**
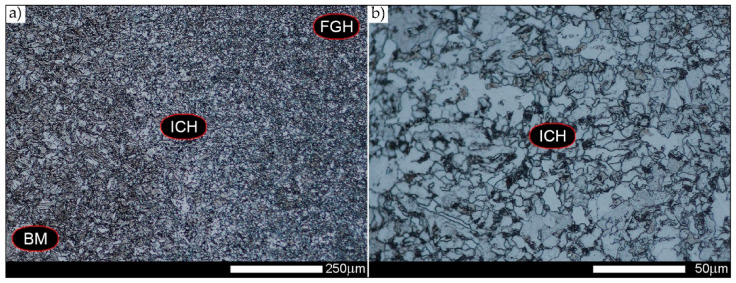
Microstructure of welded joint of Hardox 450 steel in delivery state (directly after welding)—ICHAZ zone shown in Figure 2a. (**a**) 100×; (**b**) 500×. Structurally diverse structure consisting of non-equilibrium grains of ferrite with quenching sorbite and martensite and quenching troostite. BM—parent material zone, FGH(AZ)—fine grain zone, ICH(AZ)—intercritical zone. Light microscopy, etched with 3% HNO_3_.

**Figure 13:**
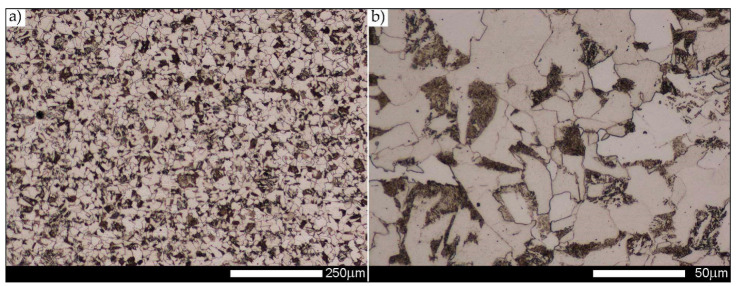
Microstructure of welded joint of Hardox 450 steel in the normalised state—BM zone shown in Figure 2b. (**a**) 100×; (**b**) 500×. Relatively homogeneous ferritic–pearlitic structure with cementite precipitates at grain boundaries. Light microscopy, etched with 3% HNO_3_.

**Figure 14:**
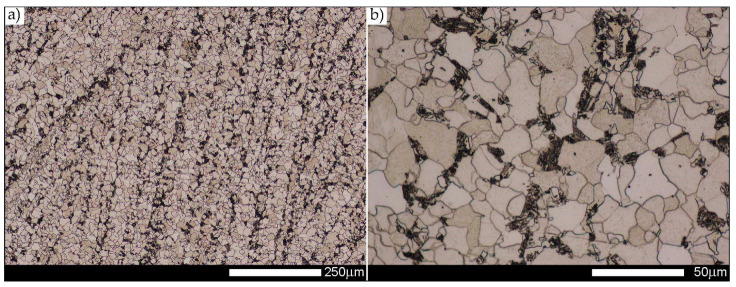
Microstructure of welded joint of Hardox 450 steel in the normalised state—WM1 zone shown in Figure 2b. (**a**) 100×; (**b**) 500×. The structure mainly consists of near-equilibrium grains of ferrite with pearlite exhibiting banded structure according to the direction of weld metal crystallisation. Some tertiary cementite precipitates are also observed at the ferrite grain boundaries. Light microscopy, etched with 3% HNO_3_.

**Figure 15:**
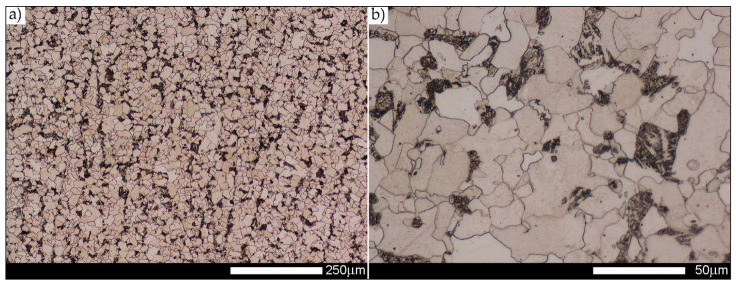
Welded joint microstructure of Hardox 450 steel in the normalised state—WM2 zone shown in Figure 2b. (**a**) 100×; (**b**) 500×. Structure consisting of near-equilibrium ferrite grains with pearlite with minor banded structure features and tertiary cementite precipitates at ferrite grain boundaries. Light microscopy, etched with 3% HNO_3_.

**Figure 16:**
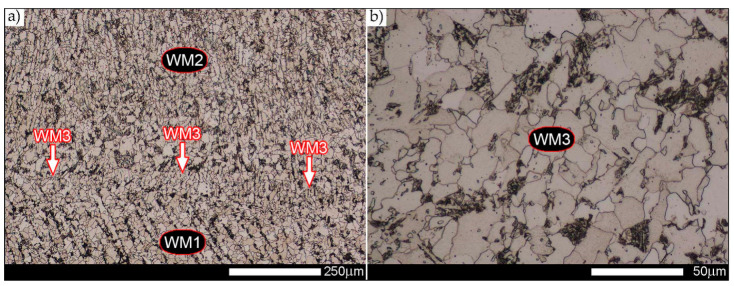
Welded joint microstructure of Hardox 450 steel in the normalised state—WM3 zone shown in Figure 2b. (**a**) 100×; (**b**) 500×. Structure of equilibrium and non-equilibrium grains of ferrite and lamellar and fine-lamellar pearlite. Tertiary cementite, often precipitated at the boundaries of ferrite grains in the form of a continuous network is also observed locally. Light microscopy, etched with 3% HNO_3_.

**Figure 17:**
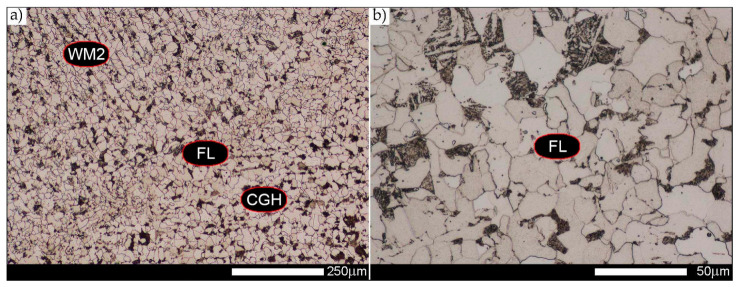
Welded joint microstructure of Hardox 450 steel in the normalised state—FL zone shown in Figure 2b. (**a**) 100×; (**b**) 500×. Structure of near-equilibrium grains of ferrite and lamellar and fine-lamellar pearlite. Tertiary cementite is also observed locally, often forming a continuous network around the ferrite grains. WM2—weld material zone 2, FL—fusion line, CGH(AZ)—coarse-grained zone. Light microscopy, etched with 3% HNO_3_.

**Figure 18:**
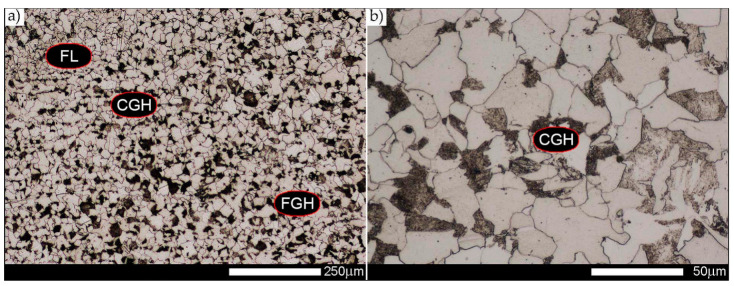
Welded joint microstructure of Hardox 450 steel in the normalised state—CGHAZ zone shown in Figure 2b. (**a**) 100×; (**b**) 500×. Structure of near-equilibrium ferrite grains with pearlite colonies. Tertiary cementite is also observed, often forming a continuous network around the ferrite grains. FL—fusion line, CGH(AZ)—coarse-grained zone, FGH(AZ)—fine-grained zone. Light microscopy, etched with 3% HNO_3_.

**Figure 19:**
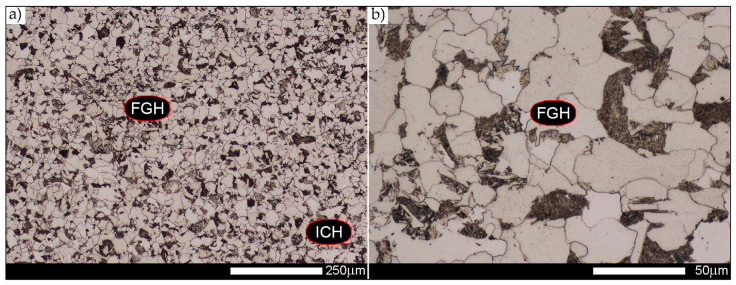
Welded joint microstructure of Hardox 450 steel in the normalised state—FGHAZ zone shown in Figure 2b. (**a**) 100×; (**b**) 500×. Structure of near-equilibrium and non-equilibrium ferrite grains with pearlite colonies. On the borders of the ferrite grains, precipitates of tertiary cementite are also observed. FGH(AZ)—fine-grained zone, ICH(AZ)—intercritical zone. Light microscopy, etched with 3% HNO_3_.

Figure 3, Figure 4, Figure 5, Figure 6, Figure 7, Figure 8, Figure 9, Figure 10, Figure 11, Figure 12, Figure 13, Figure 14, Figure 15, Figure 16, Figure 17, Figure 18 and Figure 19 show the distribution of hardness changes and microphotographs of characteristic zones of the welded joint of Hardox 450 steel, both in the delivery state and after normalising annealing. The use of the latter heat treatment for all the samples under consideration—as a preliminary operation before the subsequent heat treatments—stemmed from the complex thermal conditions during welding of the examined steel, resulting in highly diversified structural changes. This prevented a meaningful analysis of austenite grain size growth, and therefore, the normalisation treatment was intended to unify the microstructure across the welded joint to a level directly attributable to the chemical properties of Hardox 450 steel and the weld metal produced in the welding process.

The tests showed that the welding process resulted in very varied structural changes in the steel, producing broad zones of reduced hardness—see curve D in Figure 3. This course of hardness changes in the generally defined heat-affected zone is determined by the welding technology used, the value of the linear energy supplied, the parameters and conditions of welding, the chemical composition of the parent material and the weld metal, as well as the microstructure of Hardox 450 in the as-delivered state from the steel mill. In the delivery condition, Hardox 450 steel is characterised by a microstructure of quenching martensite with a slate-like morphology with areas of tempered martensite (Figure 5). The observed morphology most probably results from self-tempering processes after quenching treatments, carried out in metallurgical conditions directly after thermomechanical rolling. Therefore, in the present case, the decrease in hardness in relation to the parent material should be primarily associated with the tempering processes occurring in the broad heat-affected zone (especially in ICHAZ in Figure 2).

In the zones of the weld metal, labelled WM1, WM2, and WM3 in Figure 2, a similar structure characteristic of directional crystallisation was recorded. Overall, the microstructure of the weld metal material is dendritic and consists of non-equilibrium (acicular) ferrite—locally with Widmanstätten structure features—with troostite precipitates, nucleating mainly at the crystallisation boundaries (solidification grain boundaries)—Figure 6, Figure 7 and Figure 8.

The conclusions presented above are also confirmed by the level and uniformity of hardness in the whole zone of the weld metal—D curve in Figure 4. Figure 9 shows the microstructure of the very clearly defined, quite narrow, fusion line of the Hardox 450 welded joint. The presence of mainly sorbite and quenching troostite structures is observed along its whole length. It is worth mentioning that despite the relatively fragmented structure on the fusion line, a large growth and variable morphology of the microstructure is observed directly below it. Furthermore, it can be pointed out that due to the relatively high hardenability of the welded steel and the presence of alloying micro-additives protecting against austenite grain growth, no distinct Widmanstätten structure features are observed in the fusion zone.

Figure 10b shows an enlarged image of the microstructure in the coarse-grained zone of the CGHAZ. It shows a rather varied structure, mainly consisting of quenching sorbite and quenching martensite. In terms of morphology, martensite is characterised by a packet-like structure with very little—within the blocks covered by the grain boundaries of prior austenite—variation in crystallographic orientation. This is due to the high-temperature hardening processes taking place and the relatively low carbon content in the considered welded joint zone. Considering the course of the discussed processes at very high temperatures inducing the growth of microstructure, on the course of hardness changes, in the FL and CGHAZ zones marked on Figure 2, no distinct increase in hardness is observed, and there are even slight decreases, which are most probably caused by tempering processes (points—8.0 mm and 8.0 mm on curve D on Figure 3).

Figure 11 shows the weld area characteristic of fine-grained structures. In the zone marked as FGHAZ in Figure 2, the tested material is characterised by a fine-grained, diverse in terms of morphology, microstructure consisting of fine-lath quenching martensite, quenching sorbite, and sparse (small size) colonies of quenching troostite. Locally, single grains of non-equilibrium ferrite can also be observed.

In the intercritical cooling zone (ICHAZ area in Figure 2), the welded joint in the delivery state is characterised by a diversified and relatively size inhomogeneous microstructure (Figure 12). It can be indicated that it mainly consists of quenching sorbite with non-equilibrium ferrite, which was crystallised approximately in the direction of the parent material plastic transformation. Additionally, in the structure of the discussed zone, the presence of quenching martensite and a significant amount of quenching troostite colonies can also be detected. The observed type of structure of the welded joint intercritical zone is similar to that found in low-carbon, low-alloy steels subjected to underhardening quenching.

Figure 13, Figure 14, Figure 15, Figure 16, Figure 17, Figure 18 and Figure 19 show the microstructures of the characteristic zones of the welded joint of Hardox 450 steel in the normalised state. Based on the conducted research, it can be concluded that the applied heat treatment led to a significant unification of the microstructure of selected zones of the analysed weld joint. The above statement can mainly be applied to the CGHAZ, FGHAZ, and ICHAZ zones. Therefore, in relation to the entire heat-affected zone, further considerations on the assessment of the grain size of prior austenite were limited to the zone approximately corresponding to the FGHAZ zone, using the generalised terminology of HAZ. A detailed description of the microstructure components present in the discussed zones of the welded joint is given directly below the microphotographs.

In general, the microstructure within the whole welded joint subjected to the normalising annealing consists of non-equilibrium ferrite grains with numerous precipitates of tertiary cementite at its boundaries, as well as pearlite colonies—depending on a different carbon concentration—crystallised in various amounts. Furthermore, it is worth mentioning that the performed normalising annealing did not cause in the discussed material reduction of dendritic banding features, which was observed mainly in WM1/WM2 weld metal and resulting from directional crystallisation processes during welding.

### 3.2. Strength and Impact Tests

**Figure 20:**
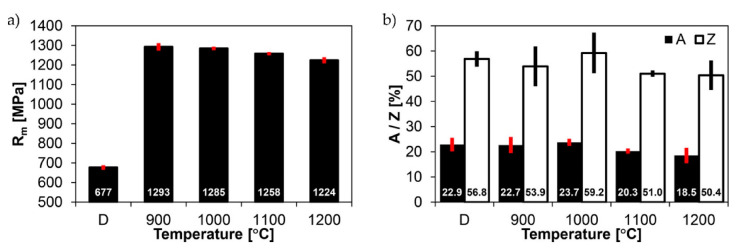
Strength tests results of welded joints of Hardox 450 steel in the delivery state and after quenching from austenitisation temperature in the range 900–1200 °C: (**a**) tensile strength, (**b**) percentage elongation after fracture and reduction of area. D—delivery state (directly after welding).

**Figure 21:**
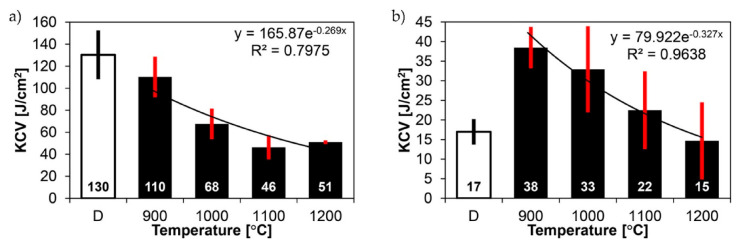
Impact test results of welded joints of Hardox 450 steel in the delivery state and after quenching from austenitising temperature in the range of 900–1200 °C: (**a**) test temperature +20 °C, (**b**) test temperature −40 °C. D—delivery state (directly after welding).

Figure 20 and Figure 21 show graphically the results of strength and impact tests of a welded joint of Hardox 450 steel in the delivery state (directly after welding) and after heat treatment procedures aimed at generating austenite grain growth in the characteristic zones of the tested material.

In the delivery state, the welded joint is characterised—relatively to the parent material in the metallurgical state—by a rather low static strength index. The obtained average value of R_m_ = 677 MPa (marked D in Figure 20a) represents approximately 85% of the alloy strength declared in the weld material technical data sheet (Table 4) and only about 50% of the parent material strength (Table 1). Significant discrepancies of the obtained indices in relation to the manufacturers’ data should be noted also in relation to the impact properties—especially at reduced temperature. The obtained impact toughness index at −40 °C, i.e., KCV = 17 J/cm^2^ (Figure 21b), strongly differs from the impact parameters of both the parent material (minimum 60.5 J/cm^2^—Table 1) and the alloy (minimum 94 J/cm^2^—Table 4). This clearly indicates that as a result of welding, the material characteristics of the brittle state were obtained (despite very satisfactory percentage elongation after fracture A and reduction of area Z indices—Figure 20b). Not going into more detailed discussion, it may be stated that the unfavourable condition of the material described above results mainly from two factors. The first of them indicates the tempering processes taking place in the heat-affected zone, resulting in a significantly reduced strength index, while the percentage elongation after fracture and reduction of area parameters are definitely high. The second factor can be associated with a very diversified (non-homogeneous in terms of morphology and grain size of prior austenite) microstructure at the fusion line, which manifests itself as a considerable decrease in impact strength parameters at sub-zero testing temperature. The observed structural and strength properties of the tested welded joint seem to confirm the correctness of the adopted research procedure, i.e., the application of normalising annealing as an operation preceding further thermal treatments, allowing us to perform a meaningful analysis of the prior austenite grain growth.

The thermal treatment, consisting of the normalisation and subsequent quenching at different temperatures in the range of 900–200 °C, did not cause significant differences in the examined welded joint of Hardox 450 steel, both in terms of static strength and indices determining the plastic properties, i.e., percentage elongation after fracture A and reduction of area Z (Figure 20). As far as the tensile strength is concerned (Figure 20a), it was noted that the austenitising before the quenching process at the highest temperature resulted in a decrease of only less than 10% of this index in relation to the samples hardened from 900 °C, which can also be assumed to be the closest to the correct austenitising temperature of the whole Hardox steel welded joint material, directly resulting from the carbon content. A similar decrease as above, of approximately 12%, can be observed in the context of reduction of area (Figure 20b). Nevertheless, this ratio of Z = 50.4% still indicates that the welded joint retains very favourable ductile properties. This statement also appears to be confirmed by the high value of percentage elongation after fracture, A = 18.5%—in relation to the tensile strength—whose reduction in relation to the heat treatment of the tested material at the lowest temperature can be estimated at approximately 20%.

Contrary to the mechanical properties determined in the static tensile test, the application of increased austenitising temperatures before quenching caused very significant changes in the impact toughness characteristics of the welded joint of the examined steel (Figure 21). The results of the research show that the welded joint quenched from 900 °C has the highest impact toughness level. This statement is valid for both impact test temperatures, i.e., +20 °C and −40 °C. Increasing the austenitising temperature by successive intervals resulted each time in a very systematic decrease of the impact strength until the welded joint material became brittle, the occurrence of which can be determined on specimens quenched from 1000 °C to 1200 °C, subjected to an impact test at −40 °C. An impact strength value lower than 35 J/cm^2^ obtained under the above-mentioned conditions, according to the literature [28], allows confirming the statement used regarding the occurrence of a brittle state.

### 3.3. Analysis of Grain Size Changes in Prior Austenite

Figure 22, Figure 23, Figure 24, Figure 25, Figure 26, Figure 27, Figure 28, Figure 29, Figure 30, Figure 31, Figure 32, Figure 33, Figure 34, Figure 35, Figure 36, Figure 37, Figure 38, Figure 39, Figure 40, Figure 41, Figure 42, Figure 43, Figure 44, Figure 45, Figure 46, Figure 47, Figure 48, Figure 49, Figure 50, Figure 51, Figure 52, Figure 53, Figure 54, Figure 55, Figure 56, Figure 57, Figure 58, Figure 59, Figure 60, Figure 61, Figure 62 and Figure 63 show microphotographs of the structures of all the more significant zones of a welded joint of Hardox 450 steel with the exposed grain boundaries of ferrite and pearlite (as a prior austenite grain boundaries). Due to the quite diverse degree of granularity of the structure, the same pattern of presentation of the structure was used in each case, based on three magnification scales. The authors believe that this approach—despite the extensive drawing documentation—significantly facilitates grain size analysis, as well as comparative assessment of the recorded structure between specific areas of the weld. Directly under each group of microphotographs, the results of grain size measurements of the prior austenite were presented in the form of graphs, taking into account the effect of austenitising temperature on all so-far discussed areas of the welded joint of Hardox steel. The final part of the study (Figure 64 and Figure 65) provides a comprehensive summary of all research results obtained.

**Figure 22:**
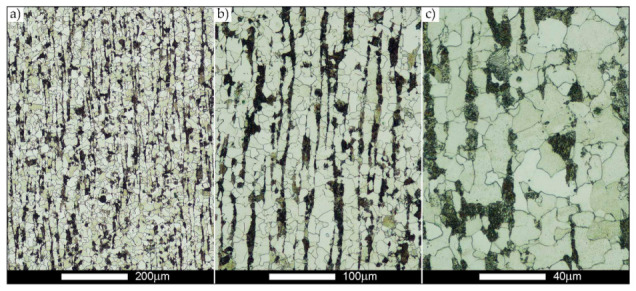
Welded joint microstructure of Hardox 450 in the delivery state (directly after welding) with revealed grain boundaries of prior austenite—BM zone shown in Figure 2. (**a**) 100×; (**b**) 200×; (**c**) 500×.

**Figure 23:**
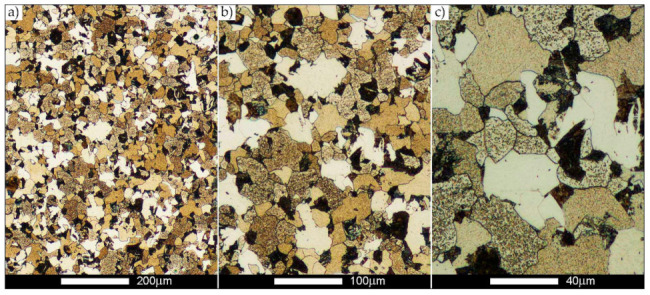
Welded joint microstructure of Hardox 450 steel in the normalised state with revealed grain boundaries of prior austenite—BM zone shown in Figure 2. (**a**) 100×; (**b**) 200×; (**c**) 500×.

**Figure 24:**
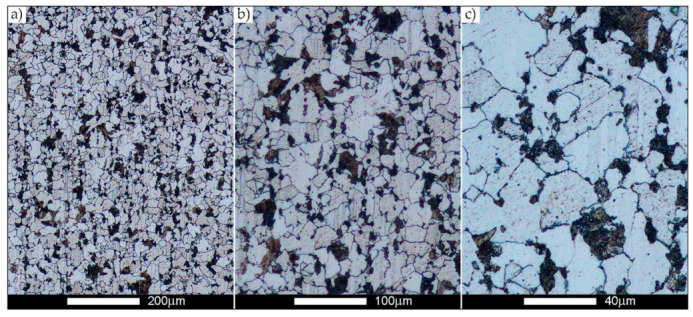
Welded joint microstructure of Hardox 450 steel with revealed grain boundaries of prior austenite, austenitised at 900 °C—BM zone shown in Figure 2. (**a**) 100×; (**b**) 200×; (**c**) 500×.

**Figure 25:**
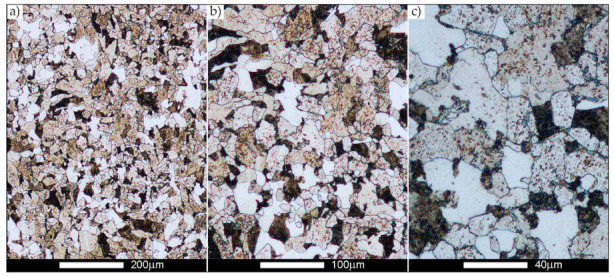
Welded joint microstructure of Hardox 450 steel with revealed grain boundaries of prior austenite, austenitised at 1000 °C—BM zone shown in Figure 2. (**a**) 100×; (**b**) 200×; (**c**) 500×.

**Figure 26:**
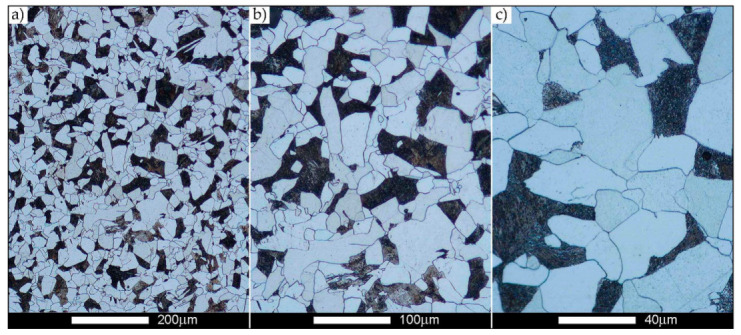
Welded joint microstructure of Hardox 450 steel with revealed grain boundaries of prior austenite, austenitised at 1100 °C—BM zone shown in Figure 2. (**a**) 100×; (**b**) 200×; (**c**) 500×.

**Figure 27:**
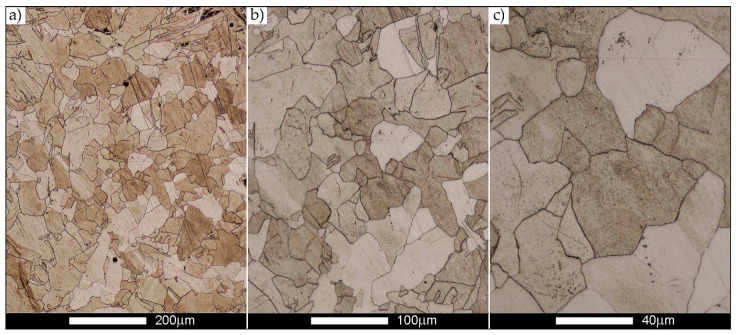
Microstructure of welded joint of Hardox 450 steel with revealed grain boundaries of prior austenite, austenitised at 1200 °C—BM zone shown in Figure 2. (**a**) 100×; (**b**) 200×; (**c**) 500×.

**Figure 28:**
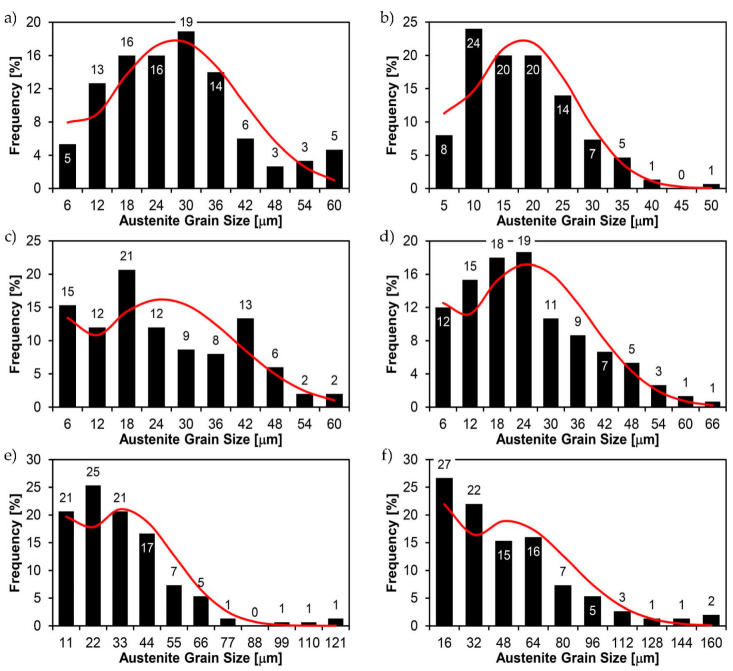
Frequency intervals and normal occurrence distributions of the determined grain sizes of prior austenite in the welded joint zone of Hardox 450 steel marked as BM in Figure 2: (**a**) normalised state; (**b**) delivery state (directly after welding); (**c**) after austenitising at 900 °C for 60 min; (**d**) after austenitising at 1000 °C for 60 min; (**e**) after austenitising at 1100 °C for 120 min; (**f**) after austenitising at 1200 °C for 120 min.

In the parent metal zone, marked BM in Figure 2, in the delivery state, the examined welded joint is characterised by a relatively small grain size, averaging 15.7 μm (Figure 22 and Figure 64a), which is a direct result of the manufacturing process of Hardox 450 steel plate. The obtained result is also well reflected by the performed normal distribution, whose maximum covers the range of 15–20 μm (Figure 28b). The application of normalising annealing causes in the discussed case an increase in the average grain size to the value of 24.7 μm (Figure 23 and N-bar in Figure 64a), which should rather be considered as a normal phenomenon, which is caused by partial recrystallisation of the structure previously subjected to thermomechanical rolling. It is also worth pointing out that the course of the maximum of the normal distribution bell curve in the range of 24–30 μm (Figure 28a) proves the correct choice of parameters and conditions of normalisation. Subjecting the zone of the parent material to additional thermal treatments, consisting of austenitising at 900 °C and 1000 °C, did not induce a clear change in the microstructural features. In the context of the thermal treatments applied, the average grain size of the prior austenite was almost identical and was 22.2 μm and 21.9 μm, respectively (Figure 24, Figure 25 and Figure 64a). A clear differentiation of the average grain sizes is observed only at the highest heat treatment temperatures. At 1100 °C and 1200 °C, average grain sizes of 28.5 μm (Figure 26 and Figure 64a) and 41.9 μm (Figure 27 and Figure 64a) were recorded, respectively. However, in both discussed cases, a significant increase of single grains up to 120 μm in size is observed (Figure 28e,f), indicating the abnormal character of prior austenite grain growth in Hardox 450 steel.

**Figure 29:**
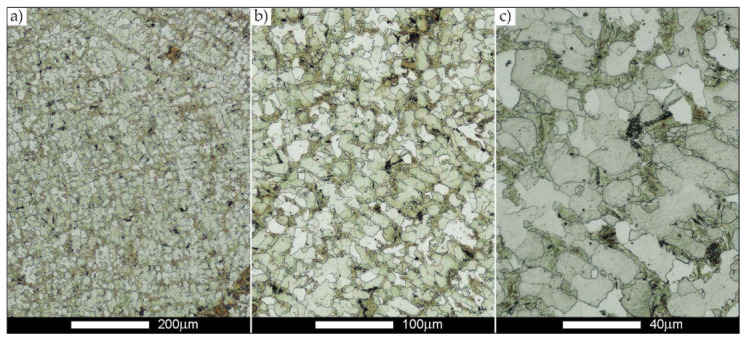
Welded joint microstructure of Hardox 450 in the delivery state (directly after welding) with revealed grain boundaries of prior austenite—WM1 zone shown in Figure 2. (**a**) 100×; (**b**) 200×; (**c**) 500×.

**Figure 30:**
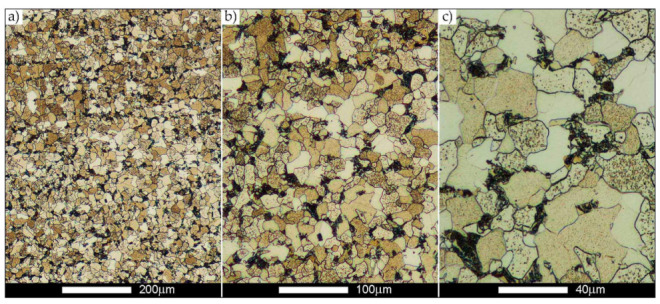
Welded joint microstructure of Hardox 450 steel in the normalised state with revealed grain boundaries of prior austenite—WM1 zone shown in Figure 2. (**a**) 100×; (**b**) 200×; (**c**) 500×.

**Figure 31:**
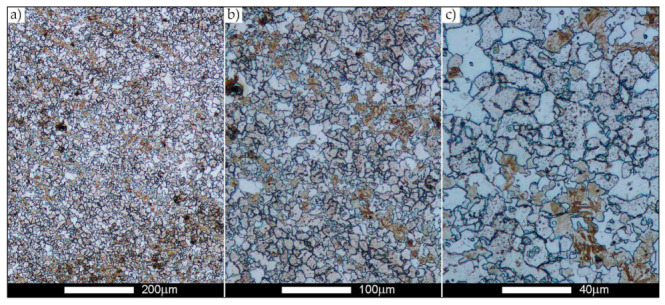
Welded joint microstructure of Hardox 450 steel with exposed grain boundaries of prior austenite, austenitised at 900 °C—WM1 zone shown in Figure 2. (**a**) 100×; (**b**) 200×; (**c**) 500×.

**Figure 32:**
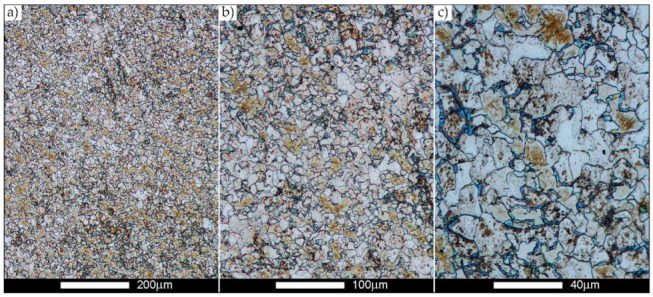
Welded joint microstructure of Hardox 450 steel with visible grain boundaries of prior austenite, austenitised at 1000 °C—WM1 zone shown in Figure 2. (**a**) 100×; (**b**) 200×; (**c**) 500×.

**Figure 33:**
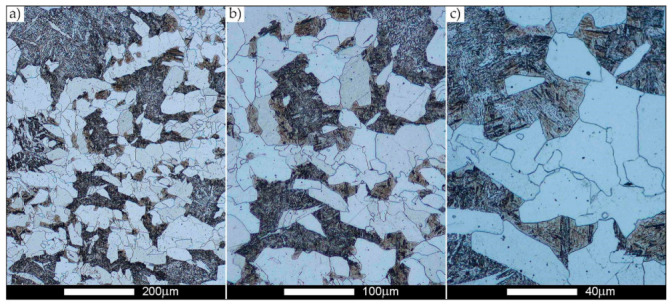
Welded joint microstructure of Hardox 450 steel with visible grain boundaries of prior austenite, austenitised at 1100 °C—WM1 zone shown in Figure 2. (**a**) 100×; (**b**) 200×; (**c**) 500×.

**Figure 34:**
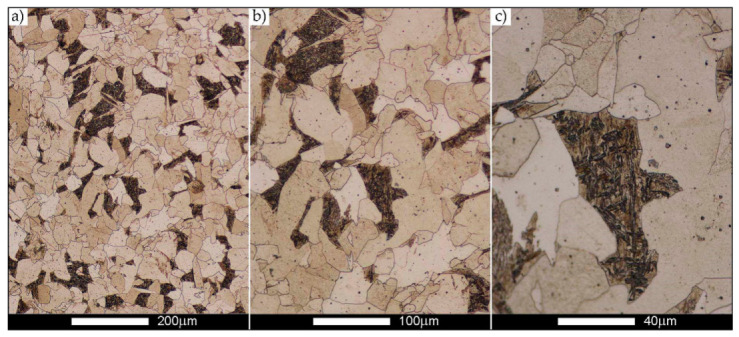
Welded joint microstructure of Hardox 450 steel with visible grain boundaries of prior austenite, austenitised at 1200 °C—WM1 zone shown in Figure 2. (**a**) 100×; (**b**) 200×; (**c**) 500×.

**Figure 35:**
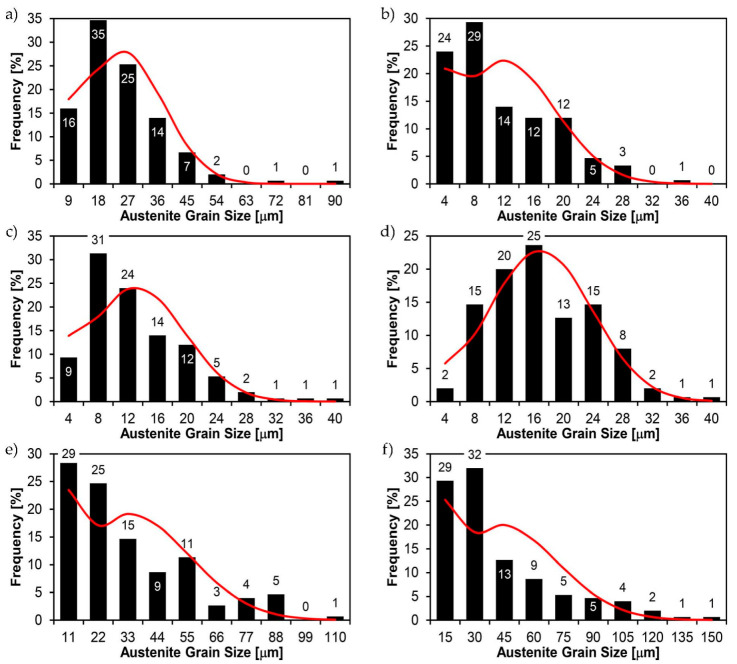
Frequency intervals and normal occurrence distributions of the determined grain sizes of prior austenite in the welded joint zone of Hardox 450 steel marked as WM1 in Figure 2: (**a**) normalised state; (**b**) delivery state (directly after welding); (**c**) after austenitising at 900 °C for 60 min; (**d**) after austenitising at 1000 °C for 60 min; (**e**) after austenitising at 1100 °C for 120 min; (**f**) after austenitising at 1200 °C for 120 min.

**Figure 36:**
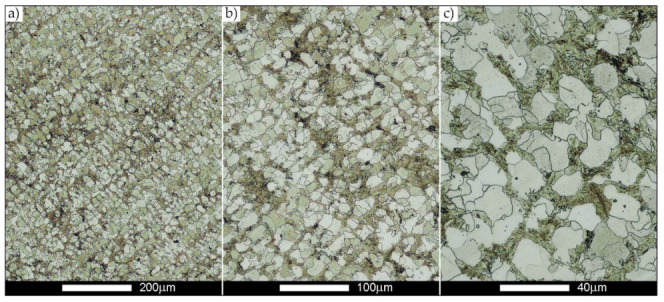
Welded joint microstructure of Hardox 450 in the delivery state (directly after welding) with revealed grain boundaries of prior austenite—WM2 zone shown in Figure 2. (**a**) 100×; (**b**) 200×; (**c**) 500×.

**Figure 37:**
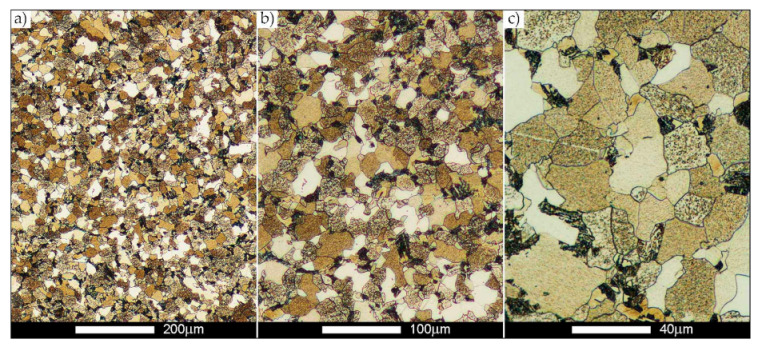
Welded joint microstructure of Hardox 450 steel in the normalised state with visible grain boundaries of prior austenite—WM2 zone shown in Figure 2. (**a**) 100×; (**b**) 200×; (**c**) 500×.

**Figure 38:**
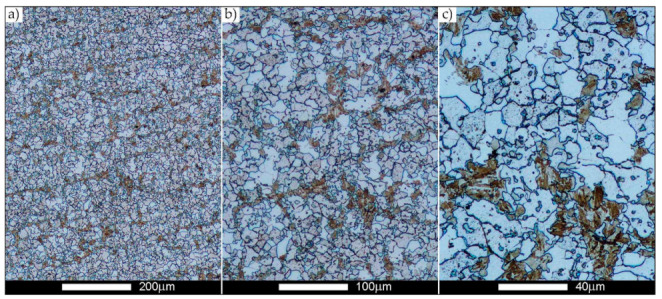
Welded joint microstructure of Hardox 450 steel with visible prior austenite grain boundaries, austenitised at 900 °C—WM2 zone shown in Figure 2. (**a**) 100×; (**b**) 200×; (**c**) 500×.

**Figure 39:**
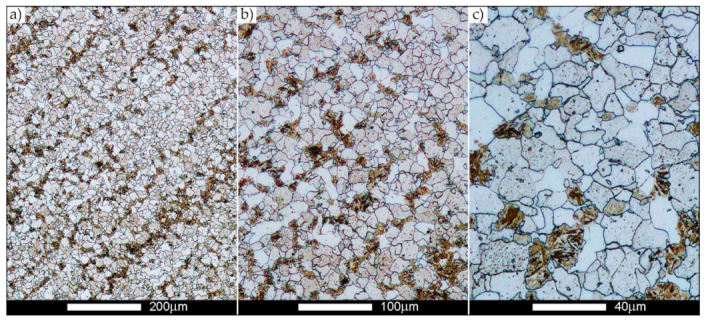
Welded joint microstructure of Hardox 450 steel with visible prior austenite grain boundaries, austenitised at 1000 °C—WM2 zone shown in Figure 2. (**a**) 100×; (**b**) 200×; (**c**) 500×.

**Figure 40:**
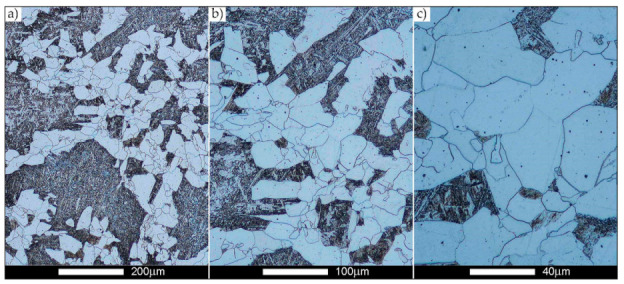
Welded joint microstructure of Hardox 450 steel with visible prior austenite grain boundaries, austenitised at 1100 °C—WM2 zone shown in Figure 2. (**a**) 100×; (**b**) 200×; (**c**) 500×.

**Figure 41:**
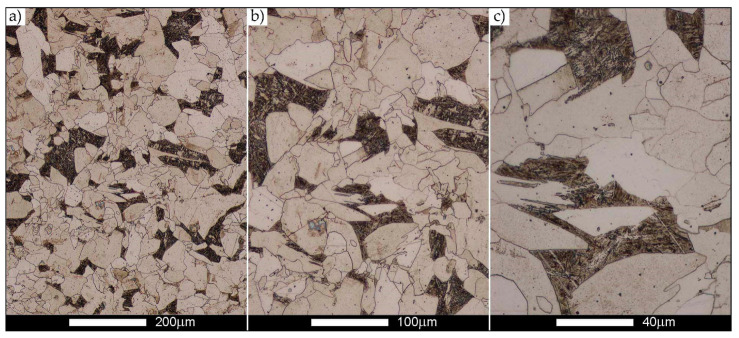
Welded joint microstructure of Hardox 450 steel with visible prior austenite grain boundaries, austenitised at 1200 °C—WM2 zone shown in Figure 2. (**a**) 100×; (**b**) 200×; (**c**) 500×.

**Figure 42:**
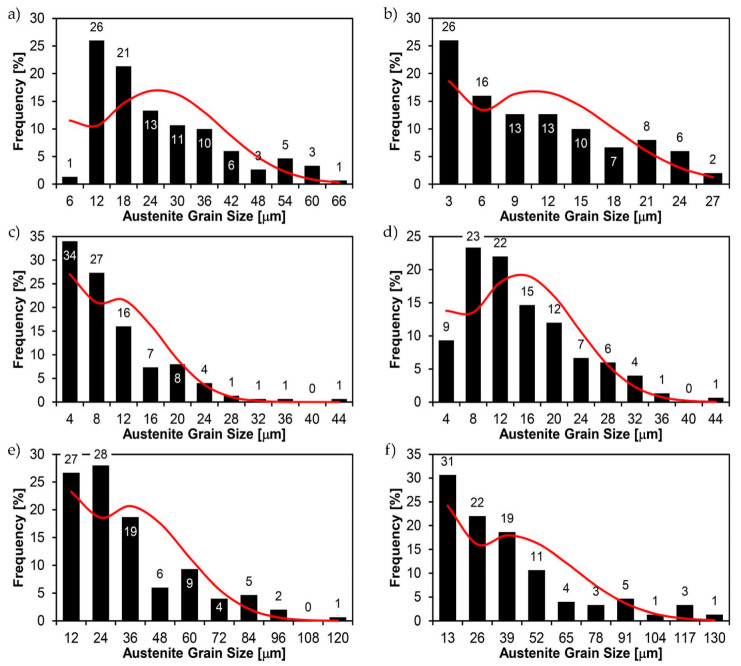
Frequency intervals and normal occurrence distributions of the determined grain sizes of prior austenite in the welded joint zone of Hardox 450 steel marked as WM2 in Figure 2: (**a**) normalised state; (**b**) delivery state (directly after welding); (**c**) after austenitising at 900 °C for 60 min; (**d**) after austenitising at 1000 °C for 60 min; (**e**) after austenitising at 1100 °C for 120 min; (**f**) after austenitising at 1200 °C for 120 min.

In the case of the weld metal, i.e., WM1 (Figure 29, Figure 30, Figure 31, Figure 32, Figure 33, Figure 34 and Figure 35) and WM2 (Figure 36, Figure 37, Figure 38, Figure 39, Figure 40, Figure 41 and Figure 42), similar values were obtained in all analysed heat treatment states. In the delivery state (directly after welding), a characteristic grain size of up to 40 µm is observed, elongated directionally to the weld axis (in line with the direction of crystallisation). Simultaneously, as a result of non-equilibrium crystallisation, a variation in the grain size of the prior austenite occurs, with grains smaller than 10 µm being the most numerous. The average grain size, for zones WM1 and WM2, is 9.7 µm and 9.3 µm, respectively. After normalisation (Figure 30, Figure 35a, Figure 37 and Figure 42a), a homogeneous structure in terms of grain shape and size is observed, which is characterised by an average prior austenite grain size of 20.4 µm and 22.7 µm (for WM1 and WM2, respectively—Figure 64b,c), as well as a bell curve reaching its maximum in the range 18–27 µm. It is also worth mentioning that due to the very high crystallisation rate, in the delivery state, the WM1 and WM2 weld material is characterised by a smaller grain size than the parent material. The heat treatment carried out proved that the average grain size in the austenitised weld material at 900 °C (corresponding approximately to the theoretical austenitisation temperature resulting from the carbon content) is 11.1 µm (WM1) and 8.3 µm (WM2). Heat treatment at 1000 °C results in an increase in the average grain size to 14.9 µm for WM1 and 12.9 µm for WM2. The average grain sizes of the WM1 and WM2 zones in the austenitised material at 1100 °C are 27.4 µm 28.7 µm, respectively, while at 1200 °C, they are 34.6 µm and 33.1 µm.

**Figure 43:**
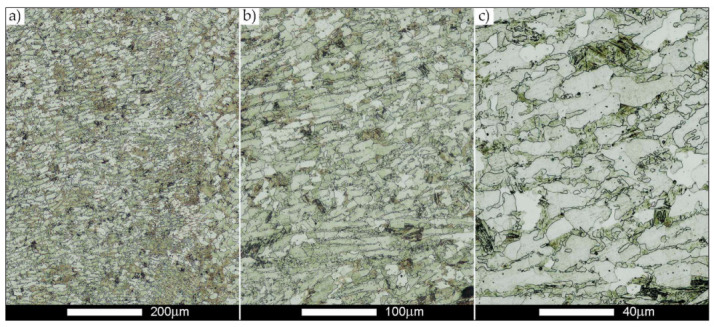
Welded joint microstructure of Hardox 450 in the delivery state (directly after welding) with visible prior austenite grain boundaries—WM3 zone shown in Figure 2. (**a**) 100×; (**b**) 200×; (**c**) 500×.

**Figure 44:**
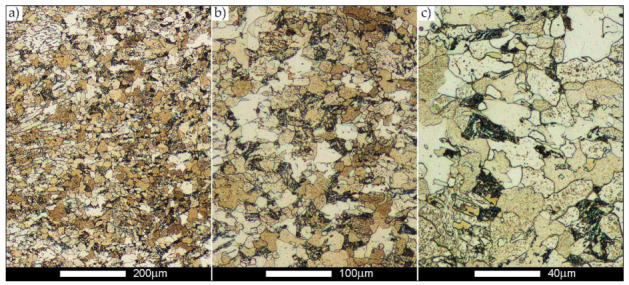
Welded joint microstructure of Hardox 450 steel in normalised state with visible prior austenite grain boundaries—WM3 zone shown in Figure 2. (**a**) 100×; (**b**) 200×; (**c**) 500×.

**Figure 45:**
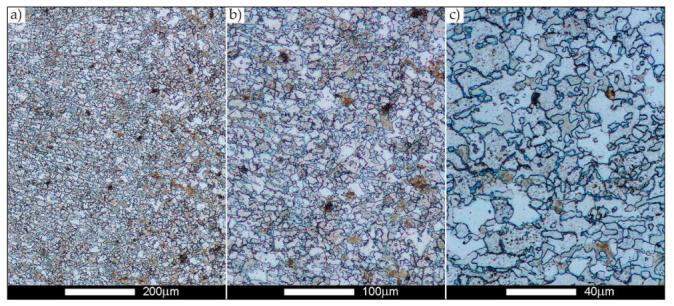
Welded joint microstructure of Hardox 450 steel with visible prior austenite grain boundaries, austenitised at 900 °C—WM3 zone shown in Figure 2. (**a**) 100×; (**b**) 200×; (**c**) 500×.

**Figure 46:**
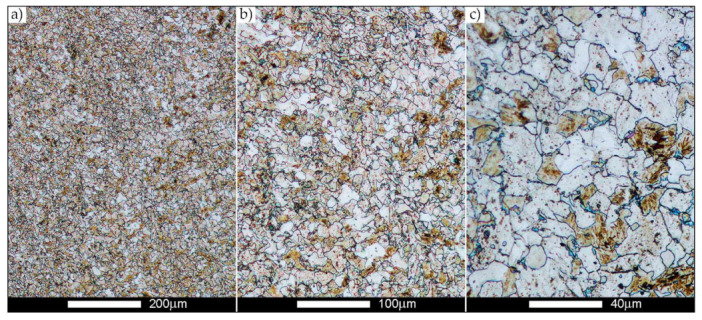
Welded joint microstructure of Hardox 450 steel with visible prior austenite grain boundaries, austenitised at 1000 °C—WM3 zone shown in Figure 2. (**a**) 100×; (**b**) 200×; (**c**) 500×.

**Figure 47:**
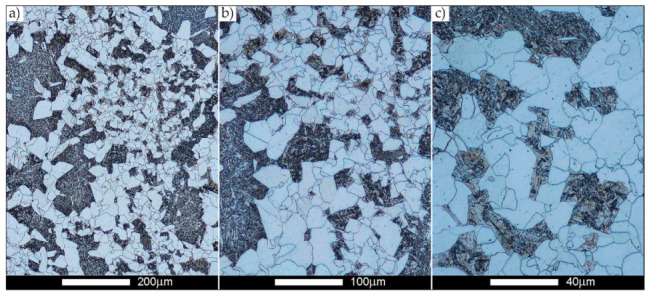
Welded joint microstructure of Hardox 450 steel with visible prior austenite grain boundaries, austenitised at 1100 °C—WM3 zone shown in Figure. 2. (**a**) 100×; (**b**) 200×; (**c**) 500×.

**Figure 48:**
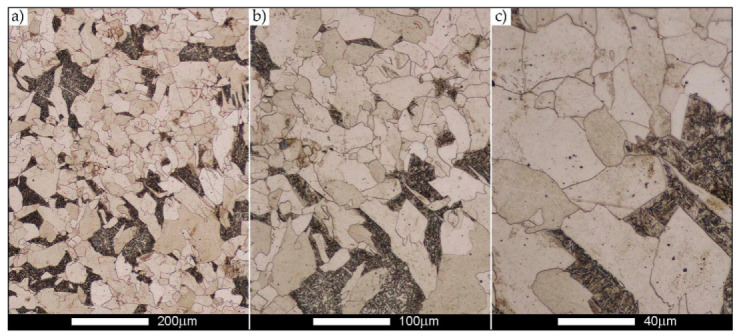
Welded joint microstructure of Hardox 450 steel with visible prior austenite grain boundaries, austenitised at 1200 °C—WM3 zone shown in Figure 2. (**a**) 100×; (**b**) 200×; (**c**) 500×.

**Figure 49:**
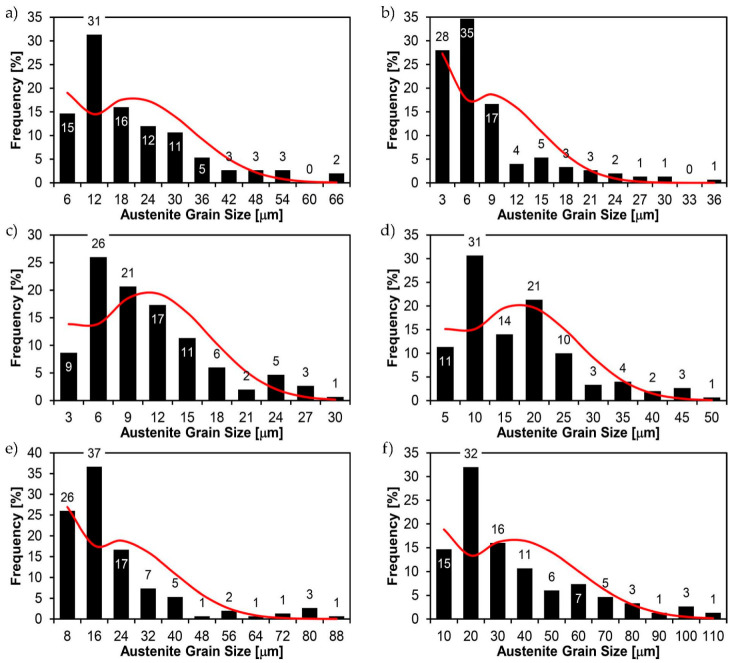
Frequency intervals and normal occurrence distributions of the determined grain sizes of prior austenite in the welded joint zone of Hardox 450 steel marked as WM3 in Figure 2: (**a**) normalised state; (**b**) delivery state (directly after welding); (**c**) after austenitising at 900 °C for 60 min; (**d**) after austenitising at 1000 °C for 60 min; (**e**) after austenitising at 1100 °C for 120 min; (**f**) after austenitising at 1200 °C for 120 min.

As for the fusion zone (line) between individual weld layers (WM3 zone in Figure 2), it can be observed that in each case considered, it was characterised by the most fragmented structure. Nevertheless, the evaluation of grain size in this zone generated quite significant interpretation difficulties due to the relatively irregular morphology of the structure. The average recorded grain size in this zone is, for the delivery state, 6.8 µm, and it is 9.6–33.1 µm for austenitisation in the temperature range 900–1200 °C (Figure 43, Figure 44, Figure 45, Figure 46, Figure 47, Figure 48, Figure 49 and Figure 64d). More specifically, austenitising at 900 and 1000 °C results in occurrence in WM3, comparable to WM1 and WM2 zones, of an average grain size of 9.6 µm and 15.0 µm, respectively. By contrast, after austenitising at 1100 °C, a local fragmentation of the structure is still observed—clearly visible in Figure 47—in which the average grain size has a value of 18.3 µm. However, the largest grains in terms of size exceed 80 µm, which clearly indicates a tendency towards growth. On the other hand, after heat treatment from 1200 °C, the average grain size is 33.1 µm, which is analogous to the WM2 zone. Furthermore, it may be indicated that the local fragmentation of the structure in the WM3 zone is mainly due to the high overcooling value during welding (lack of preheating and intermediate heating), resulting in the lack of (or limited) possibility of recrystallisation of the formed structure. This observation is confirmed by the structural properties of the WM3 zone obtained during normalisation, as a result of which the average grain size of the zone in question was 17.6 μm—Figure 44 and the N bar in Figure 64d.

**Figure 50:**
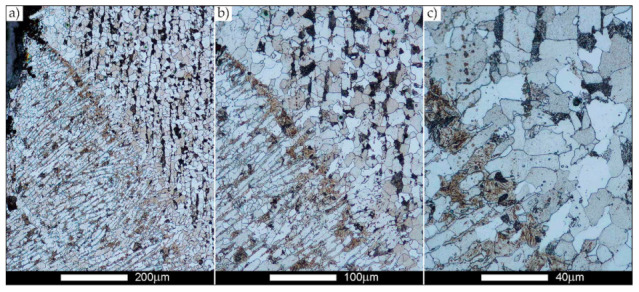
Welded joint microstructure of Hardox 450 in the delivery state (directly after welding) with visible prior austenite grain boundaries—FL zone shown in Figure 2. (**a**) 100×; (**b**) 200×; (**c**) 500×.

**Figure 51:**
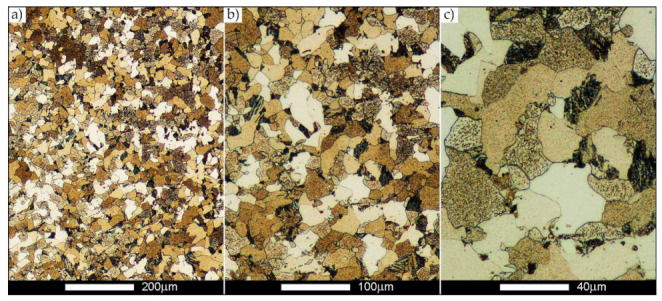
Welded joint microstructure of Hardox 450 steel in the normalised state with visible prior austenite grain boundaries—FL zone shown in Figure 2. (**a**) 100×; (**b**) 200×; (**c**) 500×.

**Figure 52:**
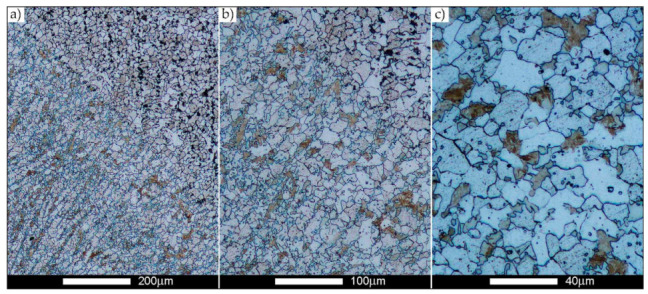
Welded joint microstructure of Hardox 450 steel with visible prior austenite grain boundaries, austenitised at 900 °C—FL zone shown in Figure 2. (**a**) 100×; (**b**) 200×; (**c**) 500×.

**Figure 53:**
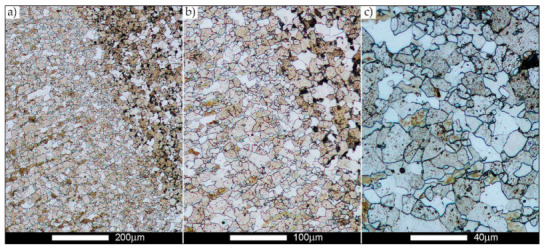
Welded joint microstructure of Hardox 450 steel with visible prior austenite grain boundaries, austenitised at 1000 °C—FL zone shown in Figure 2. (**a**) 100×; (**b**) 200×; (**c**) 500×.

**Figure 54:**
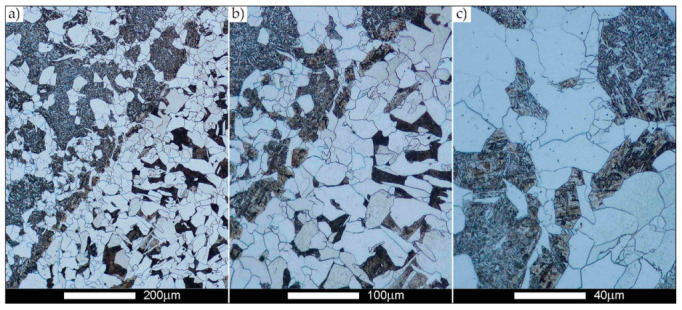
Welded joint microstructure of Hardox 450 steel with visible prior austenite grain boundaries, austenitised at 1100 °C—FL zone shown in Figure 2. (**a**) 100×; (**b**) 200×; (**c**) 500×.

**Figure 55:**
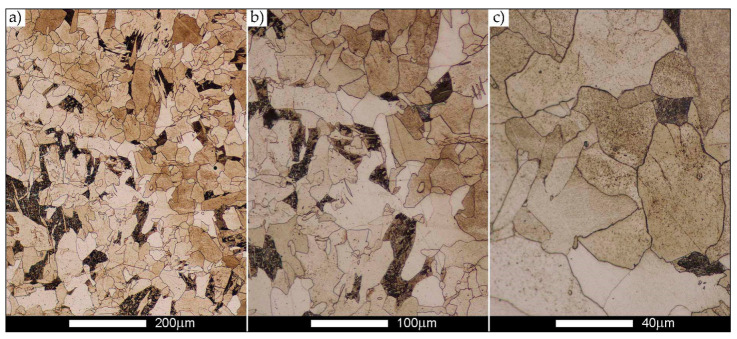
Welded joint microstructure of Hardox 450 steel with visible prior austenite grain boundaries, austenitised at 1200 °C—FL zone shown in Figure 2. (**a**) 100×; (**b**) 200×; (**c**) 500×.

**Figure 56:**
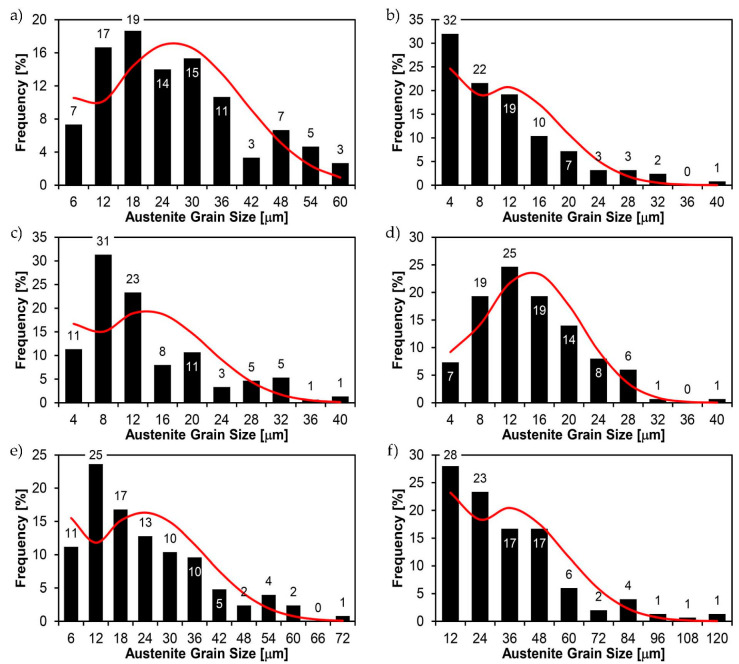
Frequency intervals and normal occurrence distributions of determined grain sizes of prior austenite in the welded joint zone of Hardox 450 steel marked as FL in Figure 2: (**a**) normalised state; (**b**) delivery state (directly after welding); (**c**) after austenitising at 900 °C for 60 min; (**d**) after austenitising at 1000 °C for 60 min; (**e**) after austenitising at 1100 °C for 120 min; (**f**) after austenitising at 1200 °C for 120 min.

Figure 50, Figure 51, Figure 52, Figure 53, Figure 54, Figure 55 and Figure 56 present microphotographs of the exhibited grain boundaries of the prior austenite and their juxtaposition in the form of frequency ranges in the FL zone (fusion line) shown in Figure 2 of the examined Hardox 450 welded joint. The crystallisation occurring under non-equilibrium conditions within the fusion line results in quite diverse grain sizes. The observations of the microstructure indicate that in this zone, there are numerous grains of size not exceeding 10 µm, as well as of much larger size of several tens of micrometres. Nevertheless, it is possible to claim that the average grain size in all analysed cases is close to that found in the WM1 and WM2 weld material. In the delivery condition (Figure 50 and Figure 56b), the average grain size of the prior austenite is 9.2 μm. Similarly to the WM3 welded material, normalisation led to a recrystallisation of the structure, inducing homogenisation and, at the same time, an increase in grain size to an average size of 23.3 μm (Figure 51 and Figure 56a). The heat treatment, consisting of austenitisation at 900 C and 1000 °C, induced a small increase in grain size compared to the delivery state, i.e., 11.9 μm (Figure 52 and Figure 56c) and 12.8 μm (Figure 53 and Figure 56d), respectively, which are still smaller values compared to the normalised state. This is a direct result of the cooling method after the annealing process—the isothermal treatment inducing a partial perlitising of the microstructure and therefore a state close to double normalisation. A more significant increase in grain size of the prior austenite is observed only at 1100 and 1200 °C. At the quoted temperatures, the average grain sizes are 20.8 µm (Figure 54 and Figure 56e) and 29.0 µm (Figure 55 and Figure 56f), respectively, and it is worth noting that an increase in the proportion of grains with sizes well above 60 μm is clearly observed at the highest temperature applied.

**Figure 57:**
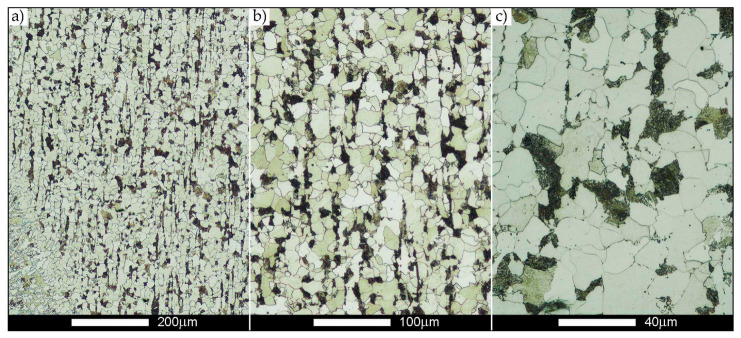
Welded joint microstructure of Hardox 450 in the delivery state (directly after welding) with visible prior austenite grain boundaries—HAZ zone. (**a**) 100×; (**b**) 200×; (**c**) 500×.

**Figure 58:**
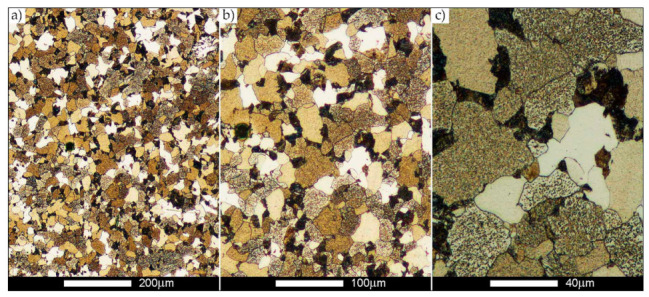
Welded joint microstructure of Hardox 450 steel in the normalised state with visible prior austenite grain boundaries—HAZ zone. (**a**) 100×; (**b**) 200×; (**c**) 500×.

**Figure 59:**
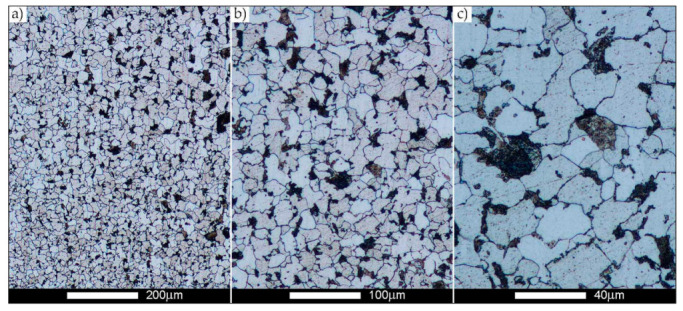
Welded joint microstructure of Hardox 450 steel with visible prior austenite grain boundaries, austenitised at 900 °C—HAZ zone. (**a**) 100×; (**b**) 200×; (**c**) 500×.

**Figure 60:**
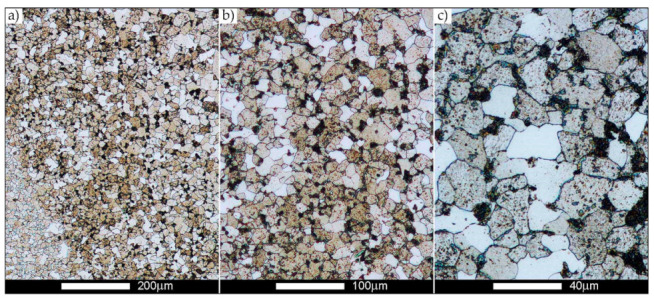
Welded joint microstructure of Hardox 450 steel with visible prior austenite grain boundaries, austenitised at 1000 °C—HAZ zone. (**a**) 100×; (**b**) 200×; (**c**) 500×.

**Figure 61:**
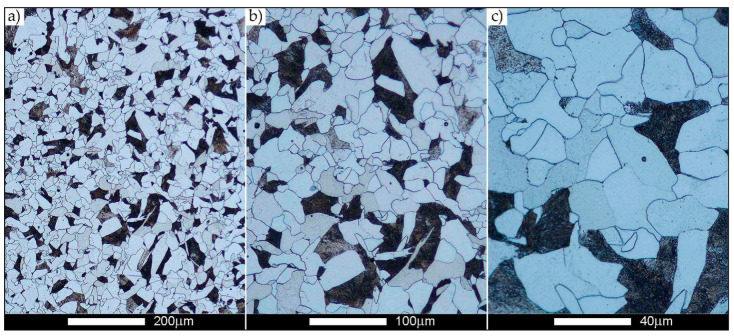
Welded joint microstructure of Hardox 450 steel with visible prior austenite grain boundaries, austenitised at 1100 °C—HAZ zone. (**a**) 100×; (**b**) 200×; (**c**) 500×.

**Figure 62:**
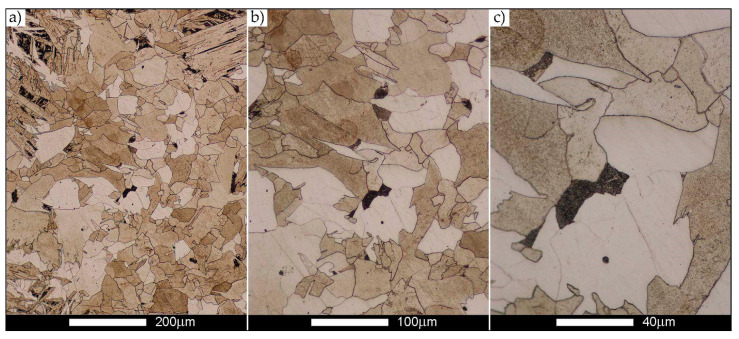
Welded joint microstructure of Hardox 450 steel with visible prior austenite grain boundaries, austenitised at 1200 °C—HAZ zone. (**a**) 100×; (**b**) 200×; (**c**) 500×.

**Figure 63:**
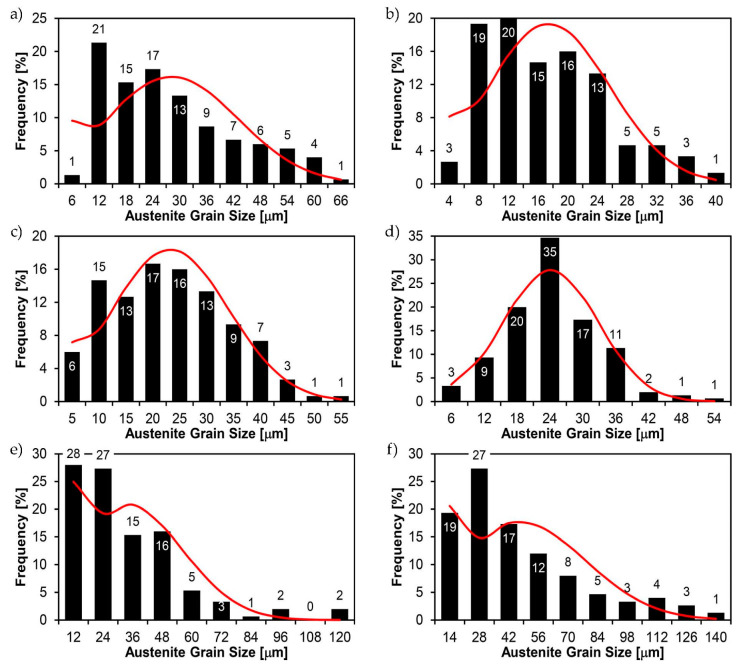
Frequency ranges and normal occurrence distributions of determined austenite grain sizes in the welded joint zone of Hardox 450 steel marked as FGH(AZ) in Figure 2: (**a**) normalised state; (**b**) delivery state (directly after welding); (**c**) after austenitising at 900 °C for 60 min; (**d**) after austenitising at 1000 °C for 60 min; (**e**) after austenitising at 1100 °C for 120 min; (**f**) after austenitising at 1200 °C for 120 min.

**Figure 64:**
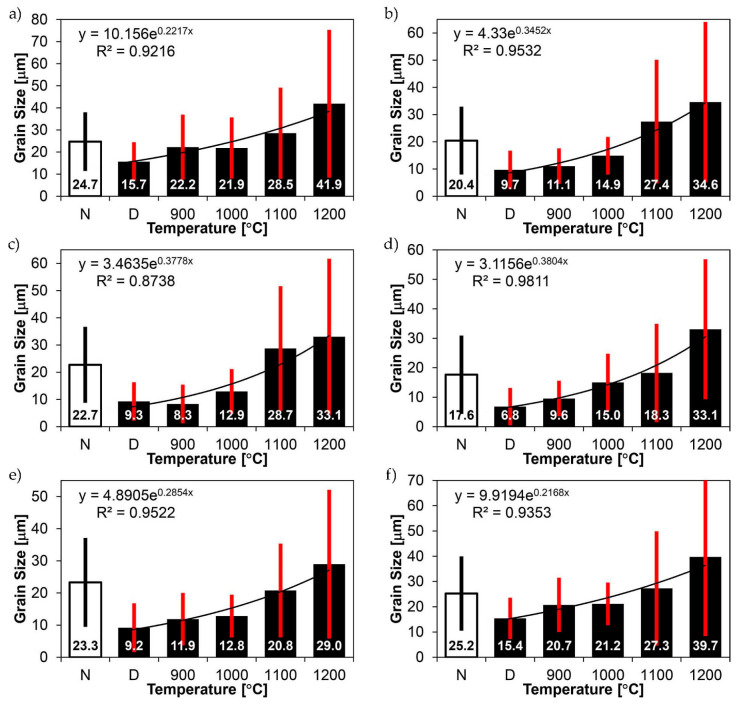
Juxtaposition of prior austenite average grain sizes as a function of temperature for individual weld zones of Hardox 450 steel shown in Figure 2: (**a**) parent material zone—BM; (**b**) weld metal zone—WM1; (**c**) weld metal zone—WM2; (**d**) weld metal zone—WM3; (**e**) fusion line—FL; (**f**) heat-affected zone—FGH(HAZ); N—normalised state, D—delivery state (directly after welding).

The last area of the Hardox 450 welded joint to be analysed is the heat-affected zone, marked as FGHAZ in Figure 2 (Figure 57, Figure 58, Figure 59, Figure 60, Figure 61, Figure 62 and Figure 63). In the delivery condition (directly after welding), the average grain size recorded is 15.4 µm (Figure 57 and Figure 64f), which corresponds very well to the grain size of the parent material of 15.7 µm (bar D in Figure 64a). In the considered case, the bell curve of the normal distribution also reaches its maximum in the range 16–20 µm. In the remaining cases considered, i.e., after normalising annealing and after austenitising at 900–1200 °C, the behaviour of the heat-affected zone was also similar to that of the Hardox 450 parent material. In the normalised condition, the average grain size is equal to 25.2 µm (Figure 58 and N bar in Figure 64f) and the normal distribution curve reaches its maximum in the interval of 24–30 µm (Figure 63a). Similar results were obtained in the material austenitised at 900 °C and 1000 °C, where the average grain size is 20.7 µm (Figure 59, Figure 63c, Figure 64f) and 21.2 µm (Figure 60, Figure 63d, Figure 64f), respectively. The response of the heat-affected zone of the tested welded joint at the highest heat treatment temperatures is also very similar to that of Hardox 450, where austenitising at 1100 °C resulted in an average grain size of 27.3 µm (Figure 61, Figure 63e, Figure 64f), while at 1200 °C, the grain size of the prior austenite increased to 39.7 µm (Figure 62, Figure 63f, Figure 64f).

**Figure 65:**
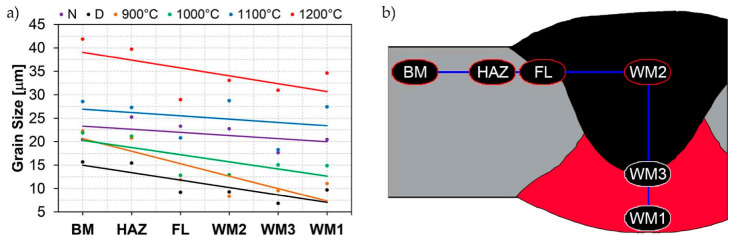
Change in average grain size of the prior austenite for different austenitising temperatures (**a**), determined in characteristic zones of the welded joint of Hardox 450 steel, running along the line schematically shown in Figure (**b**). N—normalised state, D—delivery state (directly after welding), BM—parent material, (FG)HAZ—heat-affected zone (fragmented grain), FL—fusion line, WM—melt material zones.)

Figure 65a presents changes in the average grain size as a function of the analysed zones, running along the line shown in Figure 65b. The trend line shows a steady decrease in the average grain size starting respectively from the zones of the native material and the heat-affected zone, the fusion line, and the weld metal WM2, WM3, and WM1. This shows a clear difference between the grain growth pattern in the parent and weld metal, which is characterised by a smaller average grain size and a higher point of rapid grain growth. Thus, the selection of an appropriate heat treatment for welded joints of martensitic boron alloyed steels should take into account the aforementioned differences and ensure a gradual transition from the smaller grain size in the weld metal, across the fusion line, the heat-affected zone, to the parent metal.

Based on the conducted research, the following final conclusions can be drawn:

- In the delivery state, the welded joint of Hardox 450 steel has an average tensile strength of R_m_ = 677 MPa, which is approximately 85% of the declared strength of the weld material and less than 50% of the strength of the parent material (Table 3 and Table 4 and bar D in Figure 20a).
- The structural transformations occurring during welding caused a radical reduction in the impact strength parameters of the tested joint. This conclusion applies primarily to the reduced test temperatures. Compared to the ambient temperature, in which the impact strength of the welded joint did not raise any doubts (KCV_+20_ = 130 J/cm^2^) (bar D in Figure 21a), lowering the temperature barrier to −40 °C caused in the discussed case the occurrence of the brittleness point, which was manifested by the impact strength level KCV_−40_ = 17 J/cm^2^ (bar D in Figure 21b).
- Despite very low impact strength indices at reduced temperatures and unsatisfactory strength properties, the welded joint of Hardox 450 in the delivery state was characterised by relatively high indices determining plastic properties, i.e., percentage elongation after fracture A = 22.9% and reduction of area Z = 50.4% (bars D in Figure 20b).
- The thermal treatment of the welded joint of Hardox 450 steel, consisting of normalising at 900 °C and hardening from temperatures in the range of 900–1200 °C, resulted in a significant increase in the strength (R_m_) and ductility (A and Z) indices of the tested material, with only slight differences between them being a function of the increased austenitising temperature (Figure 20). The annealing of the welded joint before hardening in the range 900–1200 °C allowed obtaining the mechanical parameters within the following ranges (respectively to temperature): R_m_ = 1293–1224 MPa (Figure 20a), A = 22.9–18.5% and Z = 56.8–50.4% (Figure 20b).
- The conducted investigations showed that for the impact test carried out at +20 °C, austenitising in the temperature range 900–1200 °C allowed obtaining the impact strength in the range of KCV_+20_ = 110–51 J/cm^2^, respectively. The influence of an increased austenitisation temperature on the impact toughness characteristics of the considered welded joint was also recorded at a reduced test temperature. In the above-described range of annealing temperatures before hardening, the obtained range of impact strength ranged from KCV_−40_ = 38–15 J/cm^2^, respectively. In this case, the brittleness point is definitely noticeable already at the austenitising temperature of 1000 °C.
- The considerations presented above, mainly concerning the impact properties of the welded joint of Hardox 450 steel, are reflected in the results of the analysis of the prior austenite grain size (Figure 64 and Figure 65). It can be stated that in the delivery state, the parent material is characterised by an average prior austenite grain size of 15.7 μm. The quenching procedures carried out from temperatures in the range of 900–1000 °C did not induce in this case any significant changes in the grain size, which can be approximated to 22 μm. It is only the quenching from higher temperatures, i.e., 1100 °C and 1200 °C, that causes a sharp increase in the average grain size, to 28.2 μm and 41.9 μm, respectively. The weld material, designated WM1 and WM2, is characterised by the finest and at the same time irregular grain size of the prior austenite. Grains smaller than 10 μm are the most numerous, with an average size of 9.7 μm (WM1) and 9.3 μm (WM2). An increase in average grain size from 11.1 to 34.6 μm for WM1 and from 8.3 to 33.1 μm for WM2 is observed in the weld material as a result of quenching processes from temperatures ranging from 900 to 1200 °C. However, it should be noted that the values obtained are slightly smaller than the corresponding values in the parent material. The fusion line between the individual weld layers (marked as WM3) is characterised by irregular grain growth, which is the result of a limited possibility of recrystallisation. The values obtained are either smaller than those obtained in the adjacent zones WM1 and WM2, or they are close to them. The most critical, from the point of view of impact properties, zone of the fusion line, marked as FL in Figure 2, is characterised by significant variation of grain size with the average value close to the one in the weld metal (WM1 and WM2). This situation is observed in this zone in all analysed cases of austenitising temperatures. On the other hand, as regards the heat-affected zone, the average grain size, both in the delivery state and in the quenched state from the temperatures ranging from 900 to 1200 °C, corresponds to analogous values in the parent material, which proves that there is no tendency for grain growth in this area as a result of increased temperature.

## Figures and Tables

**Table 1:** Chemical composition and carbon equivalents of Hardox 450 plates: PD—manufacturer’s data [1], OR—own test results, #—plate thickness, CEV—carbon equivalent according to IIW, CET—carbon equivalent according to SS-EN 1011-2, ND—no data.

Sample	C	Mn	Cr	Ni	Mo	V	Cu	CEV	CET	#
	Selected Element [wt %]							[%]		[mm]
PD	Max. 0.26	Max. 1.60	Max. 1.40	Max. 1.50	Max. 0.60	ND	ND	Max. 0.48	Max. 0.37	3.2–4.9
								Max. 0.49	Max. 0.38	5.0–9.9
								Max. 0.52	Max. 0.39	10.0–19.9
								Max. 0.60	Max. 0.41	20.0–39.9
OR	0.17	1.00	0.45	0.05	0.08	0.005	0.018	0.44	0.30	10.0
CEV = C + Mn/6 + (Cr + Mo + V)/5 + (Cu + Ni)/15; CET = C + (Mn + Mo)/10 + (Cr + Cu)/20 + Ni/40										

**Table 2:** Chemical composition of Hardox 450: PD—manufacturer’s data [1], OR—results of own research, #—plate thickness, ND—no data.

Sample	Si	P	S	Al	Ti	Nb	Co	B	#
	Selected Element [wt %]								[mm]
PD	Max. 0.70	Max. 0.025	Max. 0.010	ND	ND	ND	ND	Max. 0.005	3.2–4.9
									5.0–9.9
									10.0–19.9
									20.0–39.9
OR	0.32	0.010	0.0005	0.032	0.016	-	0.016	0.0014	10.0

**Table 3:** Mechanical properties of Hardox 450 for samples taken longitudinally to the thermo-plastic processing direction: D—delivery state, PD—manufacturer’s and distributor’s data for sheet thickness 3.2–80.0 mm [1,2], OR—own test results for samples taken from sheet thickness 30.0 mm, reported in [4], Q900-Q1200—samples hardened after austenitising at 900–1200 °C R_p0.2_—proof strength, R_m_—tensile strength, A—percentage elongation after fracture, Z—reduction of area, KCV_−40_—impact strength at −40 °C, HBW—Brinell hardness, ND—no data.

Sample	R_p0.2_	R_m_	A	Z	KCV_−40_	HBW
	[MPa]		[%]		[J/cm^2^]	
D (PD)	1250	1400–1600	10	ND	62.5	425–475
D (OR)	1106	1433	14.6	46.0	70.3	434 ± 3
Q900 (OR)	1076	1445	14.1	41.5	49.2	ND
Q1000 (OR)	1016	1413	13.2	37.3	38.3	ND
Q1100 (OR)	1006	1425	12.9	36.5	29.7	ND
Q1200 (OR)	987	1382	12.6	39.0	19.0	ND

**Table 4:** Properties of the weld deposit used to complete a welded joint of Hardox 450 steel [27]. R_p0.2_—proof strength, A_4_—percentage elongation after fracture, KCV_−40_—impact toughness at −0 °C.

Weld Deposit	C	Mn	Si	Cr	Ni	Mo	R_p0.2_	R_m_	A_4_	KCV_−40_
	Chemical Composition [wt.%]						[MPa]		[%]	[J/cm^2^]
OK Autrod 13.43 + OK Flux 10.62	0.11	1.50	0.25	0.60	2.20	0.50	700	800	21	94

**Table 5:** The framework and detailed parameters of heat treatment carried out for welded joints of Hardox 450 steels. No.1–6—samples subjected to strength and impact tests, No.7–12—samples subjected to grain growth analysis of prior austenite.

No.	Heat Treatment Scheme and Parameters		No.	Heat Treatment Scheme and Parameters	
1	D	No treatment (as welded)	2	N	Normalisation: 900 °C/1 h/Air
3	N + Q900	Normalisation: 900 °C/1 h/Air Quenching: 900 °C/1 h/H_2_O Tempering: 100 °C/2 h/Air	4	N + Q1000	Normalisation: 900 °C/1 h/Air Quenching: 1000 °C/1 h/H_2_O Tempering: 100 °C/2 h/Air
5	N + Q1100	Normalisation: 900 °C/1 h/Air Quenching: 1100 °C/2 h/H_2_O Tempering: 100 °C/2 h/Air	6	N + Q1200	Normalisation: 900 °C/1 h/Air Quenching: 1200 °C/2 h/H_2_O Tempering: 100 °C/2 h/Air
7	D + A	Austenitisation: 850 °C/10 min. Isothermal cooling: 850–650 °C/5 min/Air	8	N + A	Normalisation: 900 °C/1 h/Air Austenitisation: 900 °C/10 min Isothermal cooling: 900–650 °C/5 min/Air
9	N + A900	Normalisation: 900 °C/1 h/Air Austenitisation: 900 °C/1 h Isothermal cooling: 900–650 °C/5 min/Air	10	N + A1000	Normalisation: 900 °C/1 h/Air Austenitisation: 1000 °C/1 h Isothermal cooling: 1000–650 °C/5 min/Air
11	N + A1100	Normalisation: 900 °C/1 h/Air Austenitisation: 1100 °C/2 h Isothermal cooling: 1100–650 °C/10 min/Air	12	N + A1200	Normalisation: 900 °C/1 h/Air Austenitisation: 1200 °C/2 h Isothermal cooling: 1200–650 °C/10 min/Air

## Data Availability

The data presented in this study are available on request from the corresponding author.
